# Discovery of Antimicrobial Oligoindoles from a Cold-Seep-Derived *Halomonas* Strain

**DOI:** 10.3390/md24010016

**Published:** 2025-12-26

**Authors:** Yunchen Yan, Zhiting Li, Hongcheng Li, Junpeng Sun, Wenli Li, Fei Xiao

**Affiliations:** 1 Key Laboratory of Marine Drugs, Ministry of Education of China, School of Medicine and Pharmacy, Ocean University of China, Qingdao 266003, China; abp12138@163.com (Y.Y.); lizhiting@nwafu.edu.cn (Z.L.); lihongcheng@stu.ouc.edu.cn (H.L.); liwenli@ouc.edu.cn (W.L.); 2 Laboratory for Marine Drugs and Bioproducts, Qingdao Marine Science and Technology Center, Qingdao 266237, China; 3 State Key Laboratory for Crop Stress Resistance and High-Efficiency Production, Shaanxi Key Laboratory of Natural Products & Chemical Biology, College of Chemistry & Pharmacy, Northwest A&F University, Yangling, Xianyang 712100, China

**Keywords:** deep-sea cold seep, *Halomonas meridiana*, precursor feeding strategy, indole alkaloids, antimicrobial activity

## Abstract

Mining bioactive secondary metabolites from microorganisms originating from deep-sea cold seep holds significant potential for discovering novel drug lead compounds. In this study, three known indole derivatives (**1**–**3**) were isolated from cold-seep-derived *Halomonas meridiana* OUCLQ22-B7. Subsequently, two-new indole dimers, meribisindole A (**4**) and meribisindole B (**5**), with nine known metabolites (**6**–**14**) were obtained via indole precursor feeding strategy. The structure of these compounds was elucidated via a combination of spectroscopic methods and circular dichroism (CD) measurement. Antimicrobial assays revealed that compounds **4**, **7** and **8** exhibited potent inhibitory activity against *Fusarium oxysporum* CICC 41029 with minimal inhibitory concentrations (MICs) of 0.39−12.5 μg/mL, and compound **11** showed significant growth inhibition against *Staphylococcus aureus* CCARM 3090 with MIC value at 0.098 μg/mL.

## 1. Introduction

The unique ecosystems of deep-sea cold seep environments, characterized by methane-rich fluid emissions and unusual sulfur redox reactions, drive the microorganisms in these habitats to evolve unique metabolic pathways, which enable them to biosynthesize novel and bioactive natural products [1,2]. The identification of antifungal lipopeptide biosurfactants (surfactin CS30-1 and CS30-2) in the cold-seep derived *Bacillus* sp. CS30 has prompted increasing research on natural product discovery from deep-sea cold seep microorganisms [3]. Five new indole diketopiperazines, which displayed potent activity against the aquatic pathogen *Vibrio harveyi* (MICs =8–16 μg/mL), were isolated from deep-sea cold-seep-derived fungus *Aspergillus chevalieri* CS-122 [4]. Four unusual phenylhydrazone alkaloids (talarohydrazones A–D) were discovered from the deep-sea cold-seep-derived fungus *Talaromyces amestolkiae* HDN21-0307, among which talarohydrazone A exhibited cytotoxic activity against NCI-H446 cells (IC_50_ = 4.1 μM) as well as antibacterial activity against *Staphylococcus aureus* (MIC = 32 μg/mL) [5]. Thus, cold-seep-derived microorganisms have emerged as an important source for bioactive secondary metabolite discovery.

*Halomonas* spp., which are mainly found in saline environments, such as salt lakes, marshes and oceans, have attracted much attention due to their ability to grow rapidly in saline environments [6]. They have been developed to be effective chassis for producing various biochemicals, including bioplastics (PolyHydroxyButyrate, PHB), biopigments (β-carotene and lycopene), biofuels (propane and mandelate), amino acids and their derivatives (threonine, lysine, ectoine and cadaverine) [7]. *Halomonas* species can act as rhizobial symbionts that promote plant growth by producing plant growth hormone indole-3-acetic acid [8]. Noticeably, a few bioactive natural products have also been discovered from *Halomonas* spp. For example, three new aminophenoxazinones were isolated from marine *Halomonas* sp. via anthranilic acid feeding, which displayed antibacterial activity against *Bacillus subtilis* and *S. aureus* [9]; four new lipid siderophores (potashchelins A–D) were identified from *Halomonas* sp. MG34, and potashchelins A and D displayed inhibitory against *B. subtilis* CPCC 100029 [10]. Several reports on microbial diversity indicated that *Halomonas* is widely distributed in the cold-seep ecosystem [11], but the secondary metabolites of cold-seep-derived *Halomonas* strains remain underexploited.

Indole is a versatile signaling molecule that plays an important role in bacterial physiology, pathogenesis, animal behavior and human diseases [12]. Indole oligomers, which are widely found in plants, animals and microbes, exhibit potent bioactivities and high application values [13,14]. The notable oligoindole 3,3′-bisindolylmethane (DIM), which was first discovered from cruciferous vegetables, has been marketed as a nutraceutical as it prevents cancer and shows synergies with paclitaxel [15]. Indirubin, an indole dimer derived from plant cells, is currently used as a drug to treat chromic granulocytic leukemia [16]. Tyrian purple, mainly composed of 6,6′-dibromoindigo, is a dye extracted from sea snails and used as a biocompatible semiconductor material [17]. Several bioactive indole oligomers have also been isolated from microorganisms. For example, a new indole dimer 2,2-di(1*H*-indol-3-yl)ethyl butanoate with cytotoxic activity against the A549 lung carcinoma cell line was isolated from the marine-derived *Aeromonas* sp. CB101 [18]; an indole tetramer tetra(indol-3-yl)ethenone was isolated from a *Pseudovibrio denitrificans* strain and showed effective cytotoxic activity against the L929 mouse fibroblasts cell line [19]. Due to the good bioactivities of indole oligomers, several reports have focused on their biosynthesis. Cheng and co-workers identified a dye-decolorizing peroxidase LfDyP from the intestinal microbe *Lactobacillus fermentum*, which oxidizes indole-3-acetic acid (IAA) to indole-3-aldehyde (IAld) and indole-3-carbinol (I3C), followed by spontaneous dimerization [20]; Lee et al. and Du et al. completed the production of tyrian purple and indirubin in *E. coli* using a *Methylophaga aminisulfidivorans*-derived flavin-containing monooxygenase [21]. Therefore, exploring indole oligomers and elucidating their biosynthesis is important for the bio-manufacturing of active indole derivatives.

In this study, we investigated the secondary metabolites of deep-sea cold-seep-derived *H. meridiana* OUCLQ22-B7. Three known indole derivatives (**1**–**3**) were first isolated from the crude extract. Then precursor feeding experiments led to the activation of a series of indole oligomers, including two new (**4** and **5**) compounds and nine known indole alkaloids (**6**–**14**). Herein, we document the isolation, structure elucidation and bioactivity tests of these compounds.

## 2. Results and Discussions

**Figure 1:**
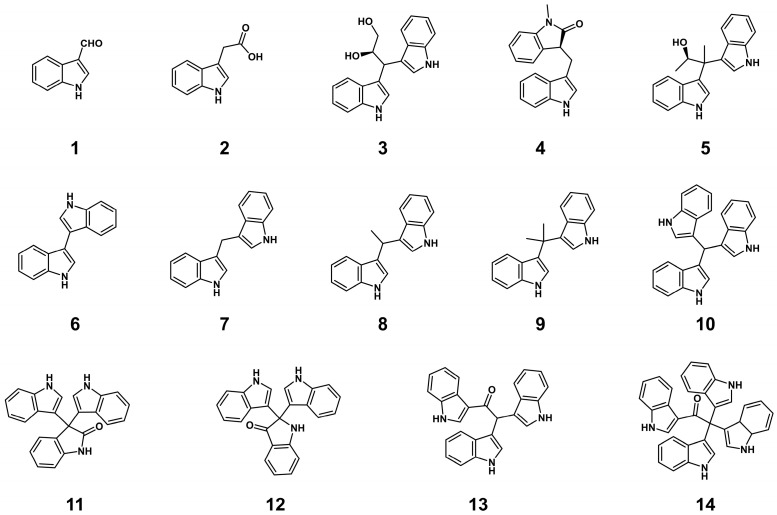
Structures of the isolated compounds (**1**–**14**) from *H. meridiana* OUCLQ22-B7.

The strain OUCLQ22-B7 was isolated from the deep-sea sediment collected at the South China Sea. The 16S rRNA gene sequence of OUCLQ22-B7 was cloned and compared against the EzTaxon-e server [22]. The phylogenetic analysis of 16S rRNA of strain OUCLQ22-B7 was shown in Figure S1. The result showed that it is highly similar to *Halomonas* 16S rRNA with 99% identity to that of *Halomonas meridiana* (GenBank accession number: ASM654012v1). Therefore, this strain was named as *H. meridiana* OUCLQ22-B7. To mine its novel metabolites, we first employed the Landy medium, CYCG medium and Lactose medium to screen for the optimal fermentation medium of *H. meridiana* OUCLQ22-B7 (Figure S2). The CYCG medium has the highest abundance of natural products (Figure S2). Subsequently, a large-scale fermentation (25 L) of *H. meridiana* OUCLQ22-B7 was performed on CYCG medium at 37 °C for 2 days. Three compounds (**1**–**3**) were isolated from the crude extract (Figure 1).

Compounds **1**–**3** were isolated as a white amorphous powder, colorless oil and a purple-red amorphous powder, respectively. Comparing the ^1^H and ^13^C NMR data (Figures S19–S24) of **1**–**3** with those reported allowed them to be identified as known compounds indole-3-carboxaldehyde (**1**) [23], indole-3-acetic acid (**2**) [24], and fusarindole B (**3**) [25].

The structure of compound **3** indicated that the deep-sea cold-seep-derived *H. meridiana* OUCLQ22-B7 has the ability to biosynthesize indole oligomers. Previous research showed that precursor feeding is an effective method to get more indole alkaloids in marine microorganisms, such as pseudboindoles A, pityriacitrin and scequinadoline A [26,27,28]. To obtain more structurally diverse indole-derived natural products, we performed precursor feeding experiments (L-Trp and indole feeding) to stimulate the production of indole-based metabolites in *H. meridiana* OUCLQ22-B7.

**Figure 2:**
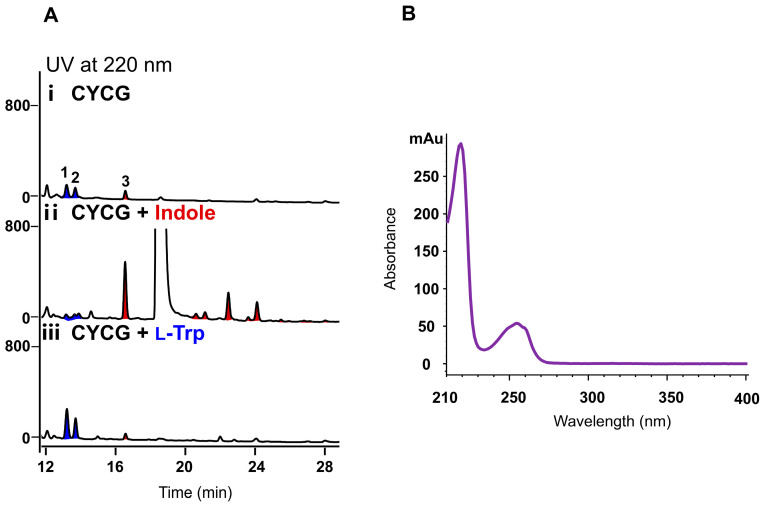
Precursor feeding experiments of *H. meridiana* OUCLQ22-B7. (**A**) HPLC analysis of the secondary metabolites of *H. meridiana* OUCLQ22-B7 in CYCG medium. (i) CYCG medium; (ii) CYCG medium supplemented with 2.5 mM indole; (iii) CYCG medium supplemented with 2.5 mM L-Trp; (**B**) UV absorbance of the oligoindoles. Indole (retention time: 18.4 min) and L-Trp (retention time: 6.9 min) were shown in Figure S3. Blue peaks represent compounds for which L-Trp is the biosynthetic precursor, while red peaks represent compounds derived from indole.

Noticeably, compared to the fermentation in CYCG medium, when supplemented with 2.5 mM L-Trp, the yield of compounds **1** and **2** increased as expected, and conversely, no obvious impact was observed for the production of compound **3**, supporting the idea that L-Trp is the direct precursor for the biosynthesis of compounds **1** and **2** (Figure 2A, panel iii). We then fed indole (2.5 mM) in the CYCG medium, and obvious effects on the metabolite profiles were detected. The production of compound **3** increased significantly (by 8.2-fold), and simultaneously a series of new peaks with similar UV spectra (Figure 2B) to indole were accumulated (Figure 2A, panel ii), indicating that indole might serve as the direct precursor for these compounds. To identify the chemical structures of these compounds, a total volume of 50 L fermentation of *H. meridiana* OUCLQ22-B7 was conducted in CYCG medium supplemented with indole. This strategy led to the isolation of six indole dimers, four indole trimers and one indole tetramer; among them two indole dimers (compounds **4** and **5**) are new compounds.

**Figure 3:**
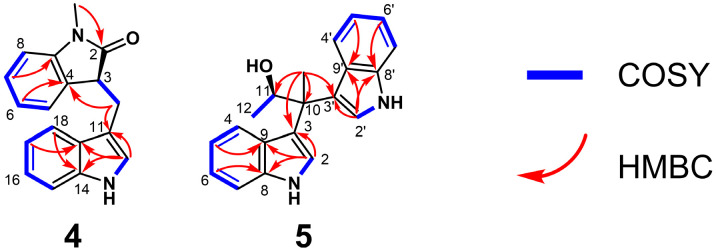
Key COSY and HMBC correlations of compounds **4** and **5**.

Compound **4** was isolated as a white amorphous powder. The HRESIMS spectrum showed a protonated molecule [M + H]^+^ at *m*/*z* 277.1338 (*calcd* for C_18_H_17_ON_2_, 277.1296), indicating the molecular formula of C_18_H_16_ON_2_, which required 12 degrees of unsaturation. The ^1^H NMR data in dimethyl sulfoxide- *d_6_* (DMSO-*d_6_*) (Table 1) showed nine olefinic protons [*δ*_H_ 6.86 (1H, t, *J* = 7.5 Hz); 7.04 (1H, m); 7.05 (1H, m); 7.07 (1H, m); 7.14 (1H, s); 7.22 (1H, br t); 7.23 (1H, br t); 7.35 (1H, d, *J* = 8.0 Hz); 8.14 (1H, d, *J* = 7.3 Hz)], one methine proton [*δ*_H_ 5.87 (1H, d, *J* = 10.6 Hz)], one methylene group [*δ*_H_ 2.97 (1H, dd, *J* = 1.3, 16.0 Hz); 3.76 (1H, dd, *J* = 10.0, 16.5 Hz)], one methyl group [*δ*_H_ 2.06 (3H, s)] and one exchangeable amino proton [*δ*_H_ 11.08 (1H, s)]. Analysis of the ^13^C NMR data of compound **4** revealed eighteen carbon signals, including one carbonyl (*δ_C_* 169.0), fourteen aromatic carbons (*δ_C_* 111.9; 116.2; 116.8; 118.6; 118.8; 121.4; 122.5; 123.5; 124.2; 124.9; 127.2; 130.8; 136.9; 143.0), one methine (*δ_C_* 57.2), one methylene (*δ_C_* 37.3) and one methyl (*δ_C_* 23.6). Detailed analysis of the 2D NMR data established the connectivity of the fragments (Figure 3). The H-15/H-16/H-17/H-18 and 13-NH/H3-12 fragments were confirmed by COSY correlations, while the key HMBC correlations from H-17 to C-19 and H-18 to C-14 established the substitution pattern. The structure of the 3-substituted indole moiety was determined by HMBC correlations from H3-12 to C-11, C-14 and C-19. The connection of C-10 to both C-3 and C-11 was established by COSY correlations between H2-10 and H-3, as well as HMBC correlations from H2-10 to C-11. Furthermore, the structure of the *ortho*-disubstituted benzene ring was confirmed based on COSY correlations among the H-5/H-6/H-7/H-8 protons and HMBC correlations from H-6 to C-4 and H-7 to C-9. Subsequently, the presence of an acetamide linkage was deduced from the HMBC correction of the methyl protons (1-CH_3_) to C-2. The N-1 atom was placed at C-9 based on NOESY correlations from 1-CH_3_ to H-9 and H3-12. Finally, the HMBC correction from H2-10 to C-8, accounting for the degrees of unsaturation, allowed for the closure of the five-membered ring between C-9 and C-8, thereby confirming the planar structure of compound **4**.

**Figure 4:**
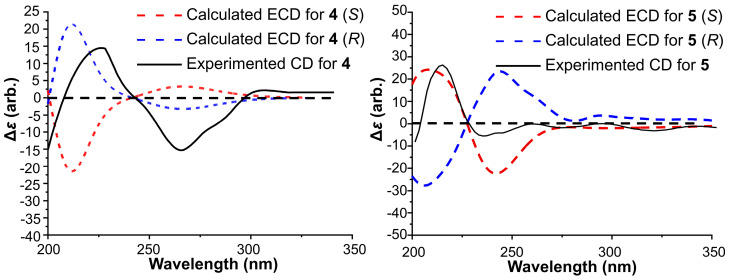
Experimental and calculated ECD spectra of compounds **4** and **5**.

To determine the absolute configuration of compound **4**, ECD calculations were performed (Figure 4). The experimental CD spectrum of **4** matched well with the calculated ECD spectrum for *R*-**4**, assigning the configuration of C-9 as *R*. Thus, compound **4** was identified as a new compound and named meribisindole A.

Compound **5** was obtained as a yellow amorphous powder. Its molecular formula was determined to be C_20_H_20_ON_2_ based on the HRESIMS peak observed at *m*/*z* 305.1652 [M + H]^+^ (*calcd* for C_20_H_21_ON_2_, 305.1609), corresponding to 12 degrees of unsaturation. The ^1^H NMR data exhibited ten aromatic protons [*δ*_H_ 6.59 (2H, dd, *J* = 7.7, 17.3 Hz); 6.85 (2H, dd, *J* = 7.8, 15.7 Hz); 7.05 (2H, dd, *J* = 8.0, 12.5 Hz); 7.25 (2H, dd, *J* = 6.2, 7.6 Hz); 7.33 (2H, d, *J* = 1.9 Hz)] suggestive of indole systems, one oxygenated methine [*δ*_H_ 4.72 (1H, dd, *J* = 6.1, 12.5 Hz)], two methyl groups [*δ*_H_ 0.95 (3H, d, *J* = 6.2 Hz); 1.74 (3H, s)] and two exchangeable amine protons [*δ*_H_ 10.74 (1H, s); 10.79 (1H, s)]. The ^13^C NMR spectrum resolved 20 carbon resonances, classified into sixteen aromatic carbons (*δ_C_* 111.0; 111.2; 117.3; 117.5; 119.9; 120.1; 120.5; 120.7; 121.0; 121.2; 122.2; 122.5; 126.4; 126.9; 136.5; 136.8), one methine (*δ_C_* 71.3), one quaternary carbon (*δ_C_* 43.3) and two methyls (*δ_C_* 19.5; 21.1). Elucidation of the planar structure was achieved through interpretation of 2D NMR correlations (Figure 3). The connectivity corresponding to the H-4–H-7 and H-1–H-2 fragments was defined by COSY correlations. Subsequently, the indole backbones were verified via HMBC correlations from H-5 to C-9, H-6 to C-8. Crucially, correlations from H-2 to C-3, C-8 and C-9 confirmed the presence of two 3-substituted indole moieties. The linkage between these fragments was established through the quaternary carbon C-10. HMBC correlations observed from the C-10 methyl protons (*δ*_H_ 1.74) to C-3, C-3′, C-10 and C-11 demonstrated that both indole rings and the methyl group were attached to C-10. The remaining substructure was resolved by COSY correlations between H3-12 and H-11. Considering the molecular formula and the chemical shift of the methine carbon (*δ_C_* 71.3), a hydroxy group was assigned to C-11. These data collectively defined the planar constitution of **5**.

The absolute configuration was addressed via ECD computational analysis (Figure 4). The experimental CD spectrum of **5** showed excellent agreement with the calculated curve for the *S*-**5**, establishing the stereochemistry at C-11 as *S*. Consequently, compound **5** was characterized as a new alkaloid and designated as meribisindole B.

Compounds **6**–**14** were isolated as yellow amorphous powders. Through comparison of the ^1^H and ^13^C NMR data with data previously reported (Figures S25–S42), they were identified as known compounds 3,3′-biindole (**6**) [29], 3,3′-diindolylmethane (**7**) [30], vibrindole A (**8**) [31], 3,3′-(1-methylethylidene)-bis-[1*H*-indole] (**9**) [32], 3,3′,3′′-mthanetriyltris-1*H*-indole (**10**) [33], 3,3-bis(1*H*-indol-3-yl)-1*H*-indol-2-one (**11**) [34], 2,2-bis(1*H*-indol-3-yl)indolin-3-one (**12**), metagenetriindole A (**13**) [35] and tetra(indol-3-yl)ethanone (**14**) [19].

Our precursor feeding experiments (Figure 2) demonstrated that, in *H. meridiana* OUCLQ22-B7, L-Trp is only the precursor of compounds **1** and **2**, whereas indole probably serves as the precursor for the bisindole, trisindole and tetraindole alkaloids **3**–**14.** It is reported that indole is an intermediate in the biosynthesis of L-Trp through the shikimic acid pathway, transforming from indole 3-glycerol phosphate; or it can be reversibly converted from L-Trp by tryptophanase (TnaA) [12]. Using the TnaA (Gene ID: 948221) from *E. coli* as a probe, we did not find any homologous protein in *H. meridiana* OUCLQ22-B7. Our results indicated that *H. meridiana* OUCLQ22-B7 lacks the reverse pathway from L-Trp to indole. In addition, for the oligomization reactions of indole derivatives, previous reports showed that the *M. aminisulfidivorans*-derived flavin-containing monooxygenase MaFMO can catalyze the hydroxylation of indole, and the hydroxylated products dimerize to indirubin by spontaneous oxidation [36], while in the intestinal microbe *L. fermentum*, a dye-decolorizing peroxidase LfDyP transforms IAA to IAId and I3C, followed by oxidation dimerization [37]. The oligomization mechanism of compounds **3**–**14** in *H. meridiana* OUCLQ22-B7 remains unknown and needs further exploration.

The antibacterial activities of compounds **1**–**14** were evaluated against three Gram-positive (*S. aureus* CCARM 3090, *Enterococcus faecalis* CCARM 5172 and *Enterococcus faecium* CCARM 5203) and three Gram-negative (*Klebsiella pneumoniae* ATCC 13883, *Escherichia coli* CCARM 1009 and *Pseudomonas aeruginosa* 15690) multidrug-resistant (MDR) bacterial strains. As shown in Table 2, compounds **5** and **11** exhibited moderate antibacterial activities against *S. aureus* CCARM3090 and *E. faecium* CCARM 5203 (MICs = 25 μg/mL), respectively, while compound **11** exhibited ~10 times stronger anti-*S. aureus* CCARM3090 activity (MIC = 0.098 μg/mL) than the positive control vancomycin (MIC = 0.78 μg/mL). This significant inhibitory activity of compound **11** against Gram-positive MDR strain *S. aureus* CCARM 3090 highlights its potential as a lead compound for developing novel antibacterial agents, which may address the growing challenges of antimicrobial resistance.

Two fungal pathogens (*C. albicans* CMCC(F) 98001 and *Fusarium oxysporum* CICC 41029) were chosen to test the antifungal activities of compounds **1**–**14**. Compounds **7**–**8** showed moderate antifungal activity against *C. albicans* CMCC(F) 98001 (MICs = 25 μg/mL); noticeably, compounds **4** and **7** exhibited ~60 times stronger anti-*F. oxysporum* CICC 41029 activities (MICs = 0.39 μg/mL) than the positive control amphotericin B (MIC = 25 μg/mL), and compound **8** exhibited 2 times stronger anti-*F. oxysporum* CICC 41029 activity (MIC = 12.5 μg/mL) than the positive control. Based on the MIC values of indole derivatives, we could conclude that, in general, indole dimers have more potent antifungal activities than indole monomers, indole trimers and the indole tetramer. Through comparison of the MICs of compounds **7**–**9**, we found that single substitution of compound **7** at C10 with a methyl group (**8**) led to a weaker activity, and double substitution of compound **7** at C10 with methyl groups (**9**) led to loss of the activity (MIC > 50 µg/mL), indicating that C10 substituents can significantly influence the antifungal effects against the plant pathogen *F. oxysporum* CICC 41029 of indole dimers. These structure–activity relationships provide a valuable starting point for the development of more potent antifungal indole-dimer agents. 

In addition, we evaluated the cytotoxic activities of compounds **1**–**14** against two tumor cell lines, A549 (human non-small cell lung cancer cell line) and HepG2 (human liver cancer cell line). Only compound **14** displayed weak cytotoxicity effect against A549 cancer cell line, with IC_50_ value of 14.12 μM (Table S3). Remarkably, compound **11**, which displayed effective anti-*S. aureus* activity, and compounds **4**, **7** and **8,** which displayed potent anti-*F. oxysporum* activity, are all non-toxic to cells at concentration of 15 μM, indicating their potential to be developed as anti-microbial drugs. 

## 3. Materials and Methods

### 3.1. Materials and General Procedures

*H. meridiana* OUCLQ22-B7 was cultured on modified LB medium (glucose 10 g/L, tryptone 10 g/L, yeast extract 5 g/L and sea-salt 33 g/L) for activation and DNA extraction. High-performance liquid chromatography (HPLC) was performed on an Agilent 1260 system (Agilent Technologies, Santa Clara, CA, USA) using a YMC-Triart C18 column (150 × 4.6 mm, 5 µm, 12 nm). The gradient Acetonitrile (ACN) −0.1% formic acid in ddH_2_O program was as follows: 10% ACN (0–5 min), 10–100% ACN (5–40 min), 100% ACN (40–50 min) and 100–10% ACN (50–56 min). The semi-preparative HPLC separation was conducted on a Hitachi Primaide system (Hitachi, Tokyo, Japan) equipped with a preparative YMC-Pack ODS-A C18 column (250 × 4.6 mm, 5 µm, 12 nm). 

The 1D and 2D NMR spectra were recorded in DMSO-*d*_6_ on a Bruker Avance III 600 (600 MHz for ^1^H and 150 MHz for ^13^C, Bruker Corporation, Billerica, MA, USA). HRESIMS data were detected on a Thermo Electron LTQ-Orbitrap XL mass spectrometer (Thermo Fisher Scientific, Waltham, MA, USA). CD spectra were acquired with a JASCO J-810 circular dichroism spectrometer (Jasco, Tokyo, Japan). All chemical reagents and solvents were purchased from standard commercial sources (Sinopharm Chemical Reagent, Shanghai, China).

### 3.2. Production and Isolation the Fermentation Products

*H. meridiana* OUCLQ22-B7 was activated on modified LB medium at 37 °C for 2 days. The stains were cultured in 250 mL Erlenmeyer flasks containing 50 mL CYCG medium (casein acid hydrolysate 6 g/L, yeast extract 2 g/L, CaCl_2_·2H_2_O 1.4 g/L, glycerol 5 mL/L and sea-salt 30 g/L, pH 7.0), Landy medium (glucose 10 g/L, L-Glutamate sodium, 4 g/L, KCl 2 g/L, KH_2_PO_4_ 1 g/L, MgSO_4_·7H_2_O 0.04 g/L, yeast extract 0.1 g/L, sea-salt 33 g/L, L-phenylalanine 2 mg/L, MnSO_4_ 5 mg/L and CuSO_4_ 0.16 mg/L, pH 7.2 ) or Lactose medium (lactose 32 g/L, peptone 8.8 g/L, yeast extract 7.5 g/L and sea-salt 33 g/L, pH 8.5), respectively, to screen for the optimal fermentation medium. Large-scale fermentation was carried out in a total volume of 25 L of CYCG medium at 37 °C for 2 days with shaking (180 rpm). For the precursor feeding experiments, CYCG medium was supplemented with sterilized 2.5 mM indole or L-Trp.

After incubation, the fermentation products were extracted twice with a double volume of EtOAc and then concentrated using rotary evaporator. The EtOAc extract of fermentation products was partitioned between 90% MeOH and *n*-hexane (1:1, *v*/*v*) to yield two residues. The aqueous MeOH layer was subjected to reversed-phase column chromatography with 10–100% MeOH to afford nine fractions (Fr.1–Fr.9). Fraction 4 (Fr.4), eluted with MeOH: H_2_O (25:75, *v*/*v*), was further purified via semi-preparative HPLC (ACN: H_2_O, 30:70, *v*/*v*, 2 mL/min) to yield compounds **1**–**3**. 

For the indole precursor feeding experiments, the fermentation products were treated as described below. The aqueous MeOH layer was subjected to reversed-phase column chromatography with 40–100% MeOH to afford six fractions (Fr.1-Fr.6). Compounds **4**–**9** were obtained from Fraction 2 (eluted with MeOH-H_2_O 50:50, *v*/*v*) by semi-preparative HPLC (ACN: H_2_O, 45:55, *v*/*v*, 2 mL/min). Compounds **10**–**14** were obtained from Fraction 5 (eluted with MeOH-H_2_O 80:20, *v*/*v*) by semi-preparative HPLC (ACN: H_2_O, 70:30, *v*/*v*, 2 mL/min).

meribisindole A (**4**): yellow powder; UV (ACN) λ_max_ 220, 280 nm; ^1^H and ^13^C NMR data, Table 1; HRESIMS *m*/*z* 277.13 [M + H]^+^, Figure S5, (calcd for C_18_H_16_ON_2_ 277.12)

meribisindole B (**5**): yellow powder; UV (ACN) λ_max_ 220, 280 nm; ^1^H and ^13^C NMR data, Table 1; HRESIMS *m*/*z* 305.16 [M + H]^+^, Figure S13, (calcd for C_20_H_20_ON_2_ 305.16)

### 3.3. Electronic Circular Dichroism (ECD) Calculations

Monte Carlo conformational searches were performed using Spartan’10 (Hongcam Software Technologies, Beijing, China) with the Merck Molecular Force Field (MMFF). Conformers with a Boltzmann population greater than 0.6% were selected for ECD calculations. Geometry optimization was subsequently conducted at the B3LYP/6-31G (d,p) level in MeOH using the CPCM polarizable conductor calculation model. Theoretical ECD spectra for compounds **4** and **5** were calculated in MeOH using time-dependent density functional theory (TD-DFT) at the B3LYP/6-31+G (d,p) level. Rotatory strengths for the first 30 excited states were calculated. Finally, ECD spectra were generated using SpecDis 1.6 (University of Würzburg, Bavaria, Germany) and GraphPad Prism 9 (University of California, Los Angeles, CA, USA) by applying Gaussian band shapes with sigma = 0.3 eV. 

### 3.4. In Vitro Antifungal Assay and Antibacterial Assay

To test the (Minimum Inhibitory Concentration) MIC values of compounds **1**–**14** against plant pathogen fungal strains *C. albicans* CMCC(F) 98001 and *F. oxysporum* CICC 41029. First, the tested strains were grown on potato dextrose agar (PDA) medium (potato 200 g/L, glucose 20 g/L and agar 20 g/L) at 30 °C for 5 days. Fresh cultures of these strains were inoculated into potato dextrose broth (PDB) medium (potato 200 g/L and glucose 20 g/L) and cultured at 30 °C for 12 h to generate a stock culture.

To measure the MIC values of compounds **1**–**14** against bacterial strains *S. aureus* CCARM 3090, *E. coli* CCARM 1009, *E. faecalis* CCARM 5172, *E. faecium* CCARM 5203, *K. pneumoniae* ATCC 13883 and *P. aeruginosa* 15690. The tested strains were grown in LB medium at 37 °C for 12 h, then adjusted to 10^6^ CFU/mL with LB medium to generate a stock culture.

Then, the cultures were adjusted and seeded into 96-well plates at 10^6^ CFU/mL per well (180 μL). The tested compounds **1**–**14** were dissolved in MeOH to obtain a stock solution (50 mg/mL). The sample stock solutions were diluted with MeOH to yield a series of concentrations (0.098, 0.19, 0.39, 0.78, 1.56, 3.12, 6.25, 12.5 and 25 μg/mL). Finally, 20 μL of the sample solution was added into each 96-well plate follower by incubation at 30 °C for 18 h. After incubation, the antifungal activity or antibacterial activity of compounds **1**–**14** was measured via a BioTek Epoch 2 microplate reader (620 nm, Agilent Technologies, Santa Clara, CA, USA). The MIC value was defined as the lowest tested compound concentration at which no bacterial or fungal growth was observed, corresponding to the first clear well in the serial dilution.

Amphotericin B (AMB) was used as a positive control for antifungal activity; Vancomycin (Van) was used as a positive control for antibacterial activity; PDB/LB medium was used as a blank control and MeOH was used as a negative control, respectively.

### 3.5. Cell Culture and Cytotoxic Assays

Human non-small cell lung cancer cell line A549 and human hepatocarcinoma cell line HepG2 (Wang Xin Lab, Hainan University, Haikou, China) were cultured in Dulbecco’s Modified Eagle Medium (DMEM) supplemented with 10% fetal bovine serum (FBS) (*v*/*v*). Cell lines were grown at 37 °C in an atmosphere with 5% CO_2_. Adherent cells (5 × 10^3^ per well) and suspension cells (8 × 10^3^ cells per well) were seeded into each 96-well plate follower by incubation at 37 °C for 24 h. Subsequently, 100 μL of the sample solution was added into each 96-well plate and then cultured at 37 °C for 72 h. After incubation, 20 μL MTT (5 mg/mL) was added into each 96-well plate and then cultured at 37 °C for 4 h, followed by treatment with 200 μL DMSO for additional 10 min with shaking. Finally, the microplate reader (560 nm) was employed to measure fluorescence intensity. The samples were tested at a concentration of 15 μM to evaluate cytotoxic activity, and if significant inhibitory effect was observed, their IC_50_ values were subsequently determined.

DMEM medium supplemented with 10% FBS was used as a blank control. Samples were dissolved in MeOH, which was used as a negative control.

## 4. Conclusions

In summary, fourteen indole derivatives, including two new indole dimers, meribisindole A (**4**) and meribisindole B (**5**), were obtained from the deep-sea cold-seep-derived strain *H. meridiana* OUCLQ22-B7. This study reveals that *H. meridiana* OUCLQ22-B7 has the ability to produce diverse indole alkaloids, and feeding indole may serve as an effective approach to obtain indole oligomers with better bioactivities. Notably, indole, rather than L-Trp, is the biosynthetic precursor of the oligoindole compounds **3**–**14** in *H. meridiana* OUCLQ22-B7, providing a starting point for further study of the tryptophan metabolic and indole oligomers biosynthetic pathways in this strain. In the antimicrobial activity tests, indole dimers **4**, **7** and **8** showed significant growth inhibitory activities against the plant pathogen *F. oxysporum* CICC 41029, whereas compound **11** displayed potent antibacterial activity against *S. aureus* CCARM 3090. The comparison of their structures and antifungal activities suggested that indole dimers have potent anti-*F. oxysporum* CICC 41029 activities and substitutes on the bridging methyl carbon influence their activities. This study explored the metabolic potential of a cold-seep-derived *Halomonas* strain that produces indole derivatives and identified promising molecules for the development of antimicrobial agents.

## Figures and Tables

**Table 1:** ^1^H NMR (400 MHz) and ^13^C NMR (150 MHz) data for compounds **4** and **5** in DMSO-*d*_6_.

Pos.	4		Pos.	5	
	*δ* *_C_*	*δ*_H_ (*J* in Hz)		*δ* *_C_*	*δ*_H_ (*J* in Hz)
2	169.0	-	2	122.2	7.33 (1H, d, *J* =1.9 Hz)
3	57.2	5.87 (1H, d, *J* = 10.6 Hz)	3	121.0	-
4	124.9	7.22 (1H, br t)	4	111.0	7.25 (1H, dd, *J* =6.2, 7.6 Hz)
5	123.5	7.04 (1H, m)	5	119.9	6.85 (1H, dd, *J* =7.8, 15.7 Hz)
6	127.2	7.23 (1H, br t)	6	117.3	6.59 (1H, dd, *J* =7.7, 17.3 Hz)
7	116.2	8.14 (1H, d, *J* = 7.3 Hz)	7	120.5	7.05 (1H, dd, *J* =8.0, 12.5 Hz)
8	143.0	-	8	126.4	-
9	130.8	-	9	136.8	-
10	37.3	2.97 (1H, dd, *J* = 1.3, 16.0 Hz) 3.76 (1H, dd, *J* = 10.0, 16.5 Hz)	10	43.3	-
11	116.8	-	11	71.3	4.72 (1H, dd, *J* =6.1, 12.5 Hz)
12	122.5	7.14 (1H, s)	12	19.5	0.95 (3H, d, *J* =6.2 Hz)
13	-	-	2′	122.5	7.51 (1H, d, *J* =2.0 Hz)
14	136.9	-	3′	121.2	-
15	111.9	7.35 (1H, d, *J* = 8.0 Hz)	4′	111.2	7.25 (1H, dd, *J* =6.2, 7.6 Hz)
16	118.8	6.86 (1H, t, *J* = 7.5 Hz)	5′	120.1	6.85 (1H, dd, *J* =7.8, 15.7 Hz)
17	121.4	7.05 (1H, m)	6′	117.5	6.59 (1H, dd, *J* =7.7, 17.3 Hz)
18	118.6	7.07 (1H, m)	7′	120.7	7.05 (1H, dd, *J* =8.0, 12.5 Hz)
19	124.2	-	8′	126.9	-
1-CH_3_	23.6	2.06 (3H, s)	9′	136.5	-
13-NH	-	11.08 (1H, s)	10-CH_3_	21.1	1.74 (3H, s)
			1-NH		10.79 (1H, s)
			1′-NH		10.74 (1H, s)

**Table 2:** Antibacterial and antifungal activities of compounds **4**, **5**, **7**, **8** and **11** against MDR bacterial strains and fungal pathogens.

MIC (µg/mL)	Strains			
	*S. aureus* CCARM 3090	*E. faecium* CCARM 5203	*C. albicans* CMCC(F) 98001	*F. oxysporum* CICC 41029
4	>50	>50	>50	0.39
5	25	25	>50	>50
7	>50	>50	25	0.39
8	>50	>50	25	12.5
11	0.098	25	>50	>50
Van	0.78	0.78		
AmB			6.25	25

Van: vancomycin as a positive control for *S. aureus* CCARM 3090 and *E. faecium* CCARM 5203. AmB: amphotericin B as a positive control for *C. albicans* CMCC(F) 98001 and *F. oxysporum* CICC 41029. The MIC values of compounds **1**–**3**, **6**, **9**–**10**, **12**–**14** against tested strains were all > 50 µg/mL. (Data were shown in Table S2).

## Data Availability

All data are contained within this article and the Supplementary Materials.

## Supplementary Materials

The following supporting information can be downloaded at https://www.mdpi.com/article/10.3390/md24010016/s1, Table S1. 16s rRNA sequence of *H. meridiana* OUCLQ22-B7; Table S2: The antibacterial and antifungal activity of compounds from *H. meridiana* OUCLQ22-B7.; Table S3: The cytotoxic activity of compound **14** against A549 cancer cell line. Figure S1: The neighbor-joining phylogenetic tree of *H. meridiana* OUCLQ22-B7 based on 16S rRNA sequence [38]; Figure S2: Optimization of the fermentation medium for *H. meridiana* OUCLQ22-B7; Figure S3 HPLC analysis the secondary metabolism of *H. meridiana* OUCLQ22-B7 in CYCG medium (i), indole standard (ii) and L-Trp standard (iii); Figure S4. UV spectrum of meribisindole A (**4**); Figure S5. HR-ESI-MS spectrum of meribisindole A (**4**); Figures S6–S11: The 1D and 2D NMR spectrum of meribisindole A (**4**) in DMSO-*d*_6_; Figure S12. UV spectrum of meribisindole B (**5**); Figure S13. HR-ESI-MS spectrum of meribisindole B (**5**); Figures S14–S18: The 1D and 2D NMR spectrum of meribisindole B (**5**) in DMSO-*d*_6_; Figures S19–S42: The ^1^H and ^13^C NMR spectrum of compounds (**1**–**3** and **6**–**14**).

## References

[1] Cong M.J., Pang X.Y., Zhao K., Song Y., Liu Y.H., Wang J.F. (2022). Deep-Sea Natural Products from Extreme Environments: Cold Seeps and Hydrothermal Vents. Mar. Drugs.

[2] Zhou Y.L., Mara P., Cui G.J., Edgcomb V.P., Wang Y. (2022). Microbiomes in the Challenger Deep slope and bottom-axis sediments. Nat. Commun..

[3] Wu S.M., Liu G., Zhou S.N., Sha Z.X., Sun C.M. (2019). Characterization of Antifungal Lipopeptide Biosurfactants Produced by Marine Bacterium *Bacillus* sp. CS30. Mar. Drugs.

[4] Yan L.H., Du F.Y., Li X.M., Yang S.Q., Wang B.G., Li X. (2023). Antibacterial Indole Diketopiperazine Alkaloids from the Deep-Sea Cold Seep-Derived Fungus *Aspergillus chevalieri*. Mar. Drugs.

[5] Wu J.J., Wang W.X., Yang Y.H., Shah M., Peng J.X., Zhou L.N., Zhang G.J., Che Q., Li J., Zhu T.J. (2024). Phenylhydrazone Alkaloids from the Deep-Sea Cold Seep Derived Fungus *Talaromyces amestolkiae* HDN21-0307. J. Nat. Prod..

[6] Zhang J., Yan X., Park H., Scrutton N.S., Chen T., Chen G.Q. (2024). Nonsterile microbial production of chemicals based on *Halomonas* spp. Curr. Opin. Biotechnol..

[7] Yan X., Wang J.L., Wen R., Chen X.Y., Chen G.Q. (2025). The halo of future bio-industry based on engineering *Halomonas*. Metab. Eng..

[8] Oliva G., Di Stasio L., Vigliotta G., Guarino F., Cicatelli A., Castiglione S. (2023). Exploring the Potential of Four Novel Halotolerant Bacterial Strains as Plant-Growth-Promoting Rhizobacteria (PGPR) under Saline Conditions. Appl. Sci..

[9] Bitzer J., Grosse T., Wang L.Z., Lang S., Beil W., Zeeck A. (2006). New aminophenoxazinones from a marine *Halomonas* sp.: Fermentation, structure elucidation, and biological activity. J. Antibiot..

[10] Li Y.H., Liu L., Zhang G.X., He N., Guo W.Q., Hong B., Xie Y.Y. (2020). Potashchelins, a Suite of Lipid Siderophores Bearing Both L-threo and L-erythro Beta-Hydroxyaspartic Acids, Acquired From the Potash-Salt-Ore-Derived Extremophile *Halomonas* sp. MG34. Front. Chem..

[11] Zhang X.Y., Wu K.Y., Han Z., Chen Z.H., Liu Z.Y., Sun Z.W., Shao L.Y., Zhao Z.L., Zhou L. (2022). Microbial diversity and biogeochemical cycling potential in deep-sea sediments associated with seamount, trench, and cold seep ecosystems. Front. Microbiol..

[12] Ma Q., Zhang X.W., Qu Y.Y. (2018). Biodegradation and Biotransformation of Indole: Advances and Perspectives. Front. Microbiol..

[13] Liu J.W., Liu A.A., Hu Y.C. (2021). Enzymatic dimerization in the biosynthetic pathway of microbial natural products. Nat. Prod. Rep..

[14] Zhang Z.L., Xu H.N., Gong C.M., Li Y.Z., Song X.M., Li Y.M., Zhang D.D., Wang R. (2025). Microorganism-Derived Bisindole Alkaloids With Anticancer Potential and Their Mechanisms: A Comprehensive Review. Chem. Biodivers..

[15] Thomson C.A., Ho E., Strom M.B. (2016). Chemopreventive properties of 3,3′-diindolylmethane in breast cancer: Evidence from experimental and human studies. Nutr. Rev..

[16] Gaboriaud-Kolar N., Vougogiannopoulou K., Skaltsounis A.L. (2015). Indirubin derivatives: A patent review (2010-present). Expert Opin. Ther. Pat..

[17] Glowacki E.D., Voss G., Leonat L., Irimia-Vladu M., Bauer S., Sariciftci N.S. (2012). Indigo and Tyrian Purple—From Ancient Natural Dyes to Modern Organic Semiconductors. Isr. J. Chem..

[18] Cai S.X., Li D.H., Zhu T.J., Wang F.P., Xiao X., Gu Q.Q. (2010). Two New Indole Alkaloids from the Marine-Derived Bacterium *Aeromonas* sp CB101. Helv. Chim. Acta.

[19] Rodrigues A.M.S., Rohée C., Fabre T., Batailler N., Sautel F., Carletti I., Nogues S., Suzuki M.T., Stien D. (2017). Cytotoxic indole alkaloids from Pseudovibrio denitrificans BBCC725. Tetrahedron Lett..

[20] Liu D., Zhang H.P., Qian J.C., Wang Y., Ren S.J., Tan R.X. (2025). Enzymatic synthesis of health-beneficial oligoindoles using peroxidase. Green Chem..

[21] Liu K.X., Chen W., Xiao Y.G., Zheng Y., Xie X.Y., Li W.L., Wang H.M., Wang H., Lin Y.N., Ye J.W. (2025). Metabolic engineering of *Halomonas* for effective production of tryptophan-derived compounds. Chem. Eng. J..

[22] Kim O.S., Cho Y.J., Lee K., Yoon S.H., Kim M., Na H., Park S.C., Jeon Y.S., Lee J.H., Yi H. (2012). Introducing EzTaxon-e: A prokaryotic 16S rRNA gene sequence database with phylotypes that represent uncultured species. Int. J. Syst. Evol. Microbiol..

[23] Yu X.D., Li L., Sun S.W., Chang A.P., Dai X.Y., Li H., Wang Y.L., Zhu H. (2021). A Cyclic Dipeptide from Marine Fungus *Penicillium chrysogenum* DXY-1 Exhibits Anti-quorum Sensing Activity. ACS Omega.

[24] Yuan Y., Zhao K., Hu Y.W., Liu Y.H., Liu Q.C., Wang J.F. (2024). Secondary Metabolites from the Mangroves-Derived *Streptomyces* sp. Scsio 41396 and Their Anti-Enzyme Activity. Chem. Nat. Compd..

[25] Thuy N.T.T., Hien H.T.M., Ha N.C., Thom L.T., Hong D.D., Thinh N.V., Dan N.T., Hoi N.D., Loan V.T., Quang H.D. (2024). Chemical constituents from the heterotrophic marine microalgae *Aurantiochytrium* sp. SC145 and their antimicrobial activities. Nat. Prod. Res..

[26] Yuan M.X., Qiu Y., Ran Y.Q., Feng G.K., Deng R., Zhu X.F., Lan W.J., Li H.J. (2019). Exploration of Indole Alkaloids from Marine Fungus Pseudallescheria boydii F44-1 Using an Amino Acid-Directed Strategy. Mar. Drugs.

[27] Chen Y.X., Xu M.Y., Li H.J., Zeng K.J., Ma W.Z., Tian G.B., Xu J., Yang D.P., Lan W.J. (2017). Diverse Secondary Metabolites from the Marine-Derived Fungus *Dichotomomyces cejpii* F31-1. Mar. Drugs.

[28] Wu D.L., Li H.J., Smith D.R., Jaratsittisin J., Xia-Ke-Er X., Ma W.Z., Guo Y.W., Dong J., Shen J., Yang D.P. (2018). Polyketides and Alkaloids from the Marine-Derived Fungus *Dichotomomyces cejpii* F31-1 and the Antiviral Activity of Scequinadoline A against Dengue Virus. Mar. Drugs.

[29] Tasch B.O.A., Antovic D., Merkul E., Müller T.J.J. (2013). One-Pot Synthesis of Camalexins and 3,3′-Biindoles by the Masuda Borylation-Suzuki Arylation (MBSA) Sequence. Eur. J. Org. Chem..

[30] Li J.T., Sun M.X., He G.Y., Xu X.Y. (2011). Efficient and green synthesis of bis(indolyl)methanes catalyzed by ABS in aqueous media under ultrasound irradiation. Ultrason. Sonochem..

[31] Bell R., Carmeli S., Sar N. (1994). Vibrindole A, a metabolite of the marine bacterium, *Vibrio parahaemolyticus*, isolated from the toxic mucus of the boxfish Ostracion cubicus. J. Nat. Prod..

[32] Shelke G.M., Rao V.K., Tiwari R.K., Chhikara B.S., Parang K., Kumar A. (2013). Bismuth triflate-catalyzed condensation of indoles with acetone. RSC Adv..

[33] Wang L.T., He X.M., Guo Y., Xu J.A., Shao S.J. (2011). Tris(indolyl)methene molecule as an anion receptor and colorimetric chemosensor: Tunable selectivity and sensitivity for anions. Org. Biomol. Chem..

[34] Fekri L.Z., Nikpassand M. (2017). 1,4-Diazabicyclo 2.2.2 octanium Diacetate under Grinding: Efficient and Eco-Friendly Process for the Synthesis of Symmetric, Unsymmetric and New Bis di(indolyl)indolin-2-one. Lett. Org. Chem..

[35] Yan X., Tang X.X., Chen L., Yi Z.W., Fang M.J., Wu Z., Qiu Y.K. (2014). Two New Cytotoxic Indole Alkaloids from a Deep-Sea Sediment Derived Metagenomic Clone. Mar. Drugs.

[36] Lee J., Kim J., Song J.E., Song W.S., Kim E.J., Kim Y.G., Jeong H.J., Kim H.R., Choi K.Y., Kim B.G. (2021). Production of Tyrian purple indigoid dye from tryptophan in *Escherichia coli*. Nat. Chem. Biol..

[37] Cheng J., Wang N.X., Yu L., Luo Y.M., Liu A.K., Tang S., Xu J.Y., Wang Y.S., Zhu J.P., Lebedev A. (2024). Molecular Insights into the One-Carbon Loss Oxidation of Indole-3-acetic Acid. ACS Catal..

[38] Tamura K., Stecher G., Kumar S. (2021). MEGA11 Molecular Evolutionary Genetics Analysis Version 11. Mol. Biol. Evol..

