# Supplementary material for: Discovery of Antimicrobial Oligoindoles from a Cold-Seep-Derived Halomonas Strain
Source: Mar Drugs. 2025 Dec 26;24(1):16. doi: 10.3390/md24010016 (PMC12842680; doi:10.3390/md24010016)
Supplement: Supplementary file 1 [file marinedrugs-24-00016-s001.zip › 20251205-marine drugs-SI.pdf]

Supporting Information

Discovery of Antimicrobial Oligoindoles from a Cold Seep-Derived *Halomonas* Strain

Yunchen Yan [1,#], Zhiting Li [1,#], Hongcheng Li [1], Junpeng Sun [1], Wenli Li [1,2,3], Fei Xiao [1,*]

[1] Key Laboratory of Marine Drugs, Ministry of Education of China, School of Medicine and Pharmacy, Ocean University of China, Qingdao 266003, China; abp12138@163.ocm (Y.Y.); lizhiting@nwafu.edu.cn (Z.L.);

[2] Laboratory for Marine Drugs and Bioproducts, Qingdao Marine Science and Technology Center, Qingdao 266237, China

[3] State Key Laboratory for Crop Stress Resistance and High-Efficiency Production, Shaanxi Key Laboratory of Natural Products & Chemical Biology, College of Chemistry & Pharmacy, Northwest A&F University, Yangling 712100, China

# Y.Y. and Z.L. contributed equally to this work.

* Correspondence: xiaofei3450@ouc.edu.cn (F.X.)

Table of contents   Page

**Table S1. 16s rRNA sequence of *H. meridiana* OUCLQ22-B7**	**S4**
**Table S2. The antibacterial and antifungal activity of compounds from *H. meridiana* OUCLQ22-B7.**	**S5**
**Table S3. The cytotoxic activity of compound 14 against A549 cancer cell line.**	**S6**
**Figure S1. The phylogenetic tree of *H. meridiana* OUCLQ22-B7 based on 16S rRNA sequence.**	**S7**
**Figure S2. Optimization of the fermentation medium for *H. meridiana***	**S8**
**Figure S3 HPLC analysis the secondary metabolism of *H. meridiana* OUCLQ22-B7 in CYCG medium (i), indole standard (ii) and L-Trp standard (iii).**	**S9**
**Figure S4. UV spectrum of meribisindole A (4)**	**S10**
**Figure S5. HR-ESI-MS spectrum of meribisindole A (4)**	**S11**
**Figure S6. ^{1}H NMR spectrum of meribisindole A (4) in DMSO-*d_6***	**S12**
**Figure S7. ^{13}C NMR spectrum of meribisindole A (4) in DMSO-*d_6***	**S13**
**Figure S8. HSQC spectrum of meribisindole A (4) in DMSO-*d_6***	**S14**
**Figure S9. ^{1}H-^{1}H COSY spectrum of meribisindole A (4) in DMSO-*d_6***	**S15**
**Figure S10. HMBC spectrum of meribisindole A (4) in DMSO-*d_6***	**S16**
**Figure S11. NOESY spectrum of meribisindole A (4) in DMSO-*d_6***	**S17**
**Figure S12. UV spectrum of meribisindole B (5)**	**S18**
**Figure S13. HR-ESI-MS spectrum of meribisindole B (5)**	**S19**
**Figure S14. ^{1}H NMR spectrum of meribisindole B (5) in DMSO-*d_6***	**S20**
**Figure S15. ^{13}C NMR spectrum of meribisindole B (5) in DMSO-*d_6***	**S21**
**Figure S16. HSQC spectrum of meribisindole B (5) in DMSO-*d_6***	**S22**
**Figure S17. ^{1}H-^{1}H COSY spectrum of meribisindole B (5) in DMSO-*d_6***	**S23**
**Figure S18. HMBC spectrum of meribisindole B (5) in DMSO-*d_6***	**S24**
**Figure S19. ^{1}H NMR spectrum of indole-3-carboxaldehyde (1) in DMSO-*d_6* (600**	**S25**
**Figure S20. DEPTQ NMR spectrum of indole-3-carboxaldehyde (1) in DMSO-**	**S26**
**Figure S21. ^{1}H NMR spectrum of indole-3-acetic acid (2) in DMSO-*d_6* (600 MHz)**	**S27**

Figure S22. DEPTQ NMR spectrum of indole-3-acetic acid (2) in DMSO-d_6 (150 **S28**

Figure S23. ^{1}H NMR spectrum of fusarindole B (3) in DMSO-d_6 (400 MHz) **S29**

Figure S24. ^{13}C NMR spectrum fusarindole B (3) in DMSO-d_6 (150 MHz) **S30**

Figure S25. ^{1}H NMR spectrum of 3,3′-biindole (6) in DMSO-d_6 (400 MHz) **S31**

Figure S26. ^{13}C NMR spectrum of 3,3′-biindole (6) in DMSO-d_6 (150 MHz) **S32**

Figure S27. ^{1}H NMR spectrum of 3,3′-diindolylmethane (7) in DMSO-d_6 (600 **S33**

Figure S28. DEPTQ NMR spectrum of 3,3′-diindolylmethane (7) in DMSO-d_6 **S34**

Figure S29. ^{1}H NMR spectrum of vibrindole A (8) in DMSO-d_6 (600 MHz) **S35**

Figure S30. DEPTQ NMR spectrum of vibrindole A (8) in DMSO-d_6 (150 MHz) **S36**

Figure S31. ^{1}H NMR spectrum of 3,3′-(1-methylethylidene)-bis-[1*H*-indole] (9) **S37**

Figure S32. DEPTQ NMR spectrum of 3,3′-(1-methylethylidene)-bis-[1*H*-indole] **S38**

Figure S33. ^{1}H NMR spectrum of 3,3′,3′′-mthanetriyltris-1*H*-indole (10) in **S39**

Figure S34. DEPTQ NMR spectrum of 3,3′,3′′-mthanetriyltris-1*H*-indole (10) in **S40**

Figure S35. ^{1}H NMR spectrum of 3,3-bis(1*H*-indol-3-yl)-1*H*-indol-2-one (11) in **S41**

Figure S36. ^{13}C NMR spectrum of 3,3-bis(1*H*-indol-3-yl)-1*H*-indol-2-one (11) in **S42**

Figure S37. ^{1}H NMR spectrum of 2,2-bis(1*H*-indol-3-yl)indolin-3-one (12) in **S43**

Figure S38. ^{13}C NMR spectrum of 2,2-bis(1*H*-indol-3-yl)indolin-3-one (12) in **S44**

Figure S39. ^{1}H NMR spectrum of metagenetriindole A (13) in DMSO-d_6 (400 MHz) **S45**

Figure S40. DEPTQ NMR spectrum of metagenetriindole A (13) in DMSO-d_6 (150 MHz) **S46**

Figure S41. ^{1}H NMR spectrum of metagenetriindole A (14) in DMSO-d_6 (400 MHz) **S47**

Figure S42. ^{13}C NMR spectrum of metagenetriindole A (14) in DMSO-d_6 (150 MHz) **S48**

Reference **S49**

Table S1. 16s rRNA sequence of *H. meridiana* OUCLQ22-B7.

AGAGTTTGATCATGGCTCAGATTGAACGCTGGCGGCAGGCCTAACACATGCAAGTC<br>GAGCGGTAACAGATCCAGCTTGCTGGATGCTGACGAGCGGCGGACGGGTGAGTAA<br>TGCATAGGAATCTGCCCGATAGTGGGGGATAACCTGGGGAAACCCAGGCTAATAC<br>CGCATACGTCCTACGGGAGAAAGGGGGCTCCGGCTCCCGCTATGGGATGAGCCTA<br>TGTCGGATTAGCTAGTTGGTGAGGTAACGGCTCACCAAGGCCACGATCCGTAGCTG<br>GTCTGAGAGGATGATCAGCCACATCGGGACTGAGACACGGCCCGAACTCCTACGG<br>GAGGCAGCAGTGGGGAATATTGGACAATGGGGGCAACCCTGATCCAGCCATGCCG<br>CGTGTGTGAAGAAGGCCCTCGGGTTGTAAAGCACTTTCAGCGAGGAAGAACGCCT<br>AGCGGTTAATACCCGCTAGGAAAGACATCACTCGCAGAAGAAGCACCGGCTAACT<br>CCGTGCCAGCAGCCGCGGTAATACGGAGGGTGCAAGCGTTAATCGGAATTACTGG<br>GCGTAAAGCGCGCGTAGGTGGCTTGATAAGCCGGTTGTGAAAGCCCCGGGCTCAA<br>CCTGGGAACGGCATCCGGAACTGTCAAGCTAGAGTGCAGGAGAGGAAGGTAGAA<br>TTCCCGGTGTAGCGGTGAAATGCGTAGAGATCGGGAGGAATACCAGTGGCGAAGG<br>CGGCCTTCTGGACTGACACTGACACTGAGGTGCGAAAGCGTGGGTAGCAAACAGG<br>ATTAGATACCCTGGTAGTCCACGCCGTAAACGATGTCGACCAGCCGTTGGGTGCCT<br>AGCGCACTTTGTGGCGAAGTTAACGCGATAAGTCGACCGCCTGGGGAGTACGGCC<br>GCAAGGTTAAAACTCAAATGAATTGACGGGGGCCCGCACAAGCGGTGGAGCATGT<br>GGTTTAATTCGATGCAACGCGAAGAACCTTACCTACTCTTGACATCCTGCGAATTTG<br>GTAGAGATACCTTAGTGCCTTCGGGAACGCAGAGACAGGTGCTGCATGGCTGTCGT<br>CAGCTCGTGTTGTGAAATGTTGGGTTAAGTCCCGTAACGAGCGCAACCCTTGTCCTT<br>ATTTGCCAGCGCGTAATGGCGGGAACTCTAAGGAGACTGCCGGTGACAAACCGGA<br>GGAAGGTGGGGACGACGTCAAGTCATCATGGCCCTTACGAGTAGGGCTACACACG<br>TGCTACAATGGTCGGTACAAAGGGTTGCCAACTCGCGAGAGTGAGCCAATCCCGA<br>AAAGCCGATCTCAGTCCGGATCGGAGTCTGCAACTCGACTCCGTGAAGTCGGAATC<br>GCTAGTAATCGTGGATCAGAATGCCACGGTGAATACGTTCCCGGGCCTTGTACACA<br>CCGCCCGTCACACCATGGGAGTGGACTGCACCAGAAGTGGTTAGCCTAACGCAAG<br>AGGGCGATCACCACGGTGTGGTTCATGACTGGGGTGAAGTCGTAACAAGGTAGCC<br>GTAGGGGAACCTGCGGCTGGATCACCT

Table S2. The antibacterial and antifungal activities of compounds (**1-14**) isolated from *H. meridiana* OUCLQ22-B7.

**MIC(μg/mL)**	**Strains**							
	S. aureus CCARM 3090	*E. faecium* CCARM 5203	*E. faecalis* *CCARM* 5172	*P. aeruginosa* 15690	*E. coli* *CCARM* 1009	*K. pneumoniae* ATCC 13883	*C. albicans* CMCC(F) 98001	*F. oxysporum* CICC 41029
**1**	>50	>50	>50	>50	>50	>50	>50	>50
**2**	>50	>50	>50	>50	>50	>50	>50	>50
**3**	>50	>50	>50	>50	>50	>50	>50	>50
**4**	>50	>50	>50	>50	>50	>50	>50	0.39
**5**	25	25	>50	>50	>50	>50	>50	>50
**6**	>50	>50	>50	>50	>50	>50	>50	>50
**7**	>50	>50	>50	>50	>50	>50	25	0.39
**8**	>50	>50	>50	>50	>50	>50	25	12.5
**9**	>50	>50	>50	>50	>50	>50	>50	>50
**10**	>50	>50	>50	>50	>50	>50	>50	>50
**11**	0.098	25	>50	>50	>50	>50	>50	>50
**12**	>50	>50	>50	>50	>50	>50	>50	>50
**13**	>50	>50	>50	>50	>50	>50	>50	>50
**14**	>50	>50	>50	>50	>50	>50	>50	>50
**Tet**				12.5	1.56	12.5		
**Van**	0.78	0.78	0.78					
**AmB**							6.25	6.25

Tet: tetracycline as positive control for *E. faecalis* CCARM 5172, *P. aeruginosa* 15690, *E. coli* CCARM 1009 and *K. pneumoniae* ATCC 13883.

Van: vancomycin as positive control for *S. aureus* CCARM 3090 and *E. faecium* CCARM 5203.

AmB: Amphotericin B as positive control for *C. albicans* CMCC(F) 98001 and *F. oxysporum* CICC 41029.

Table S3. The cytotoxic activity of compound **14** against A549 cancer cell line.

**Compound**	**IC_{50} (±SD) [μM]**
	**A549**
**14**	14.12 ± 1.53

Figure S1. The phylogenetic tree of *H. meridiana* OUCLQ22-B7 based on 16S rRNA sequence.

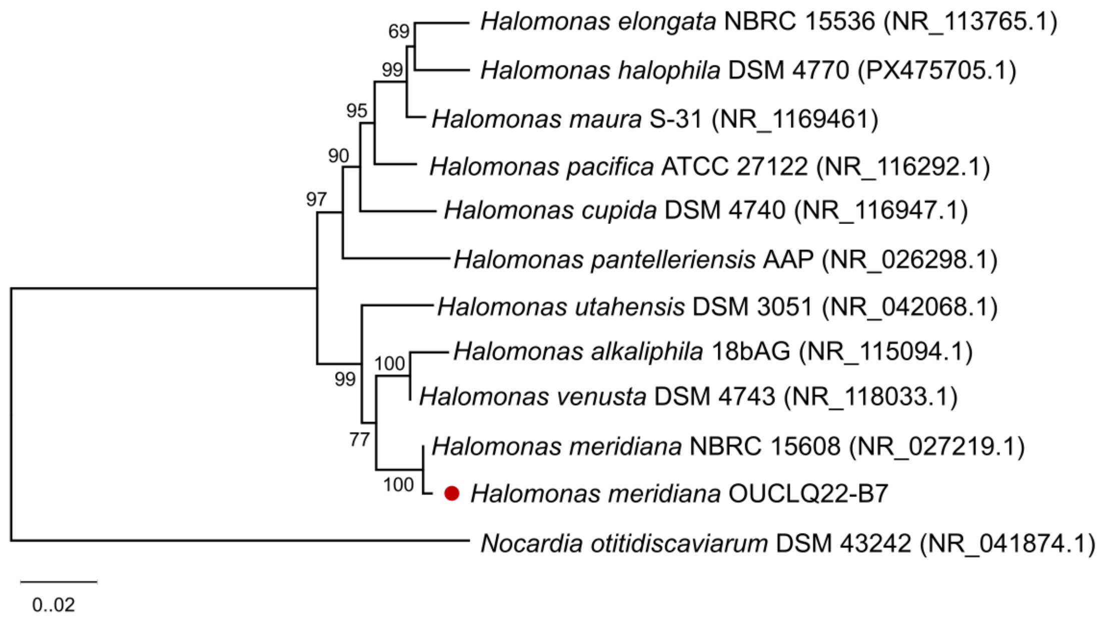

The 16S rRNA sequence of other strains was download from NCBI database. Phylogenetic tree was constructed using the Neighbor-Joining (bootstrapped with 500 replicates) in MEGA software[1].

.

Figure S2. Optimization of the fermentation medium for *H. meridiana* OUCLQ22-B7. (**i**) The HPLC profile of *H. meridiana* OUCLQ22-B7 fermented in CYCG medium. (**ii**) The HPLC profile of *H. meridiana* OUCLQ22-B7 fermented in Lactose medium. (**iii**) HPLC profile of *H. meridiana* OUCLQ22-B7 fermented in Landy medium.

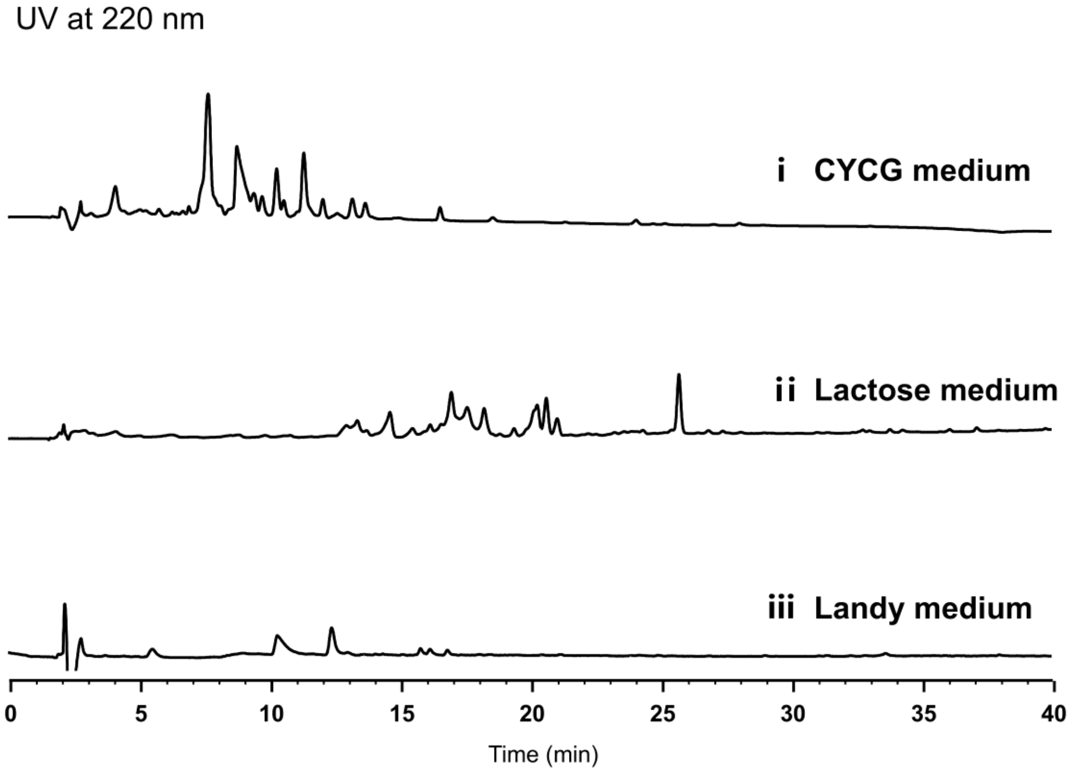

Figure S3. HPLC analysis of the fermentation metabolites of *H. meridiana* OUCLQ22-B7 in CYCG medium (i), indole standard (ii) and L-Trp standard (iii).

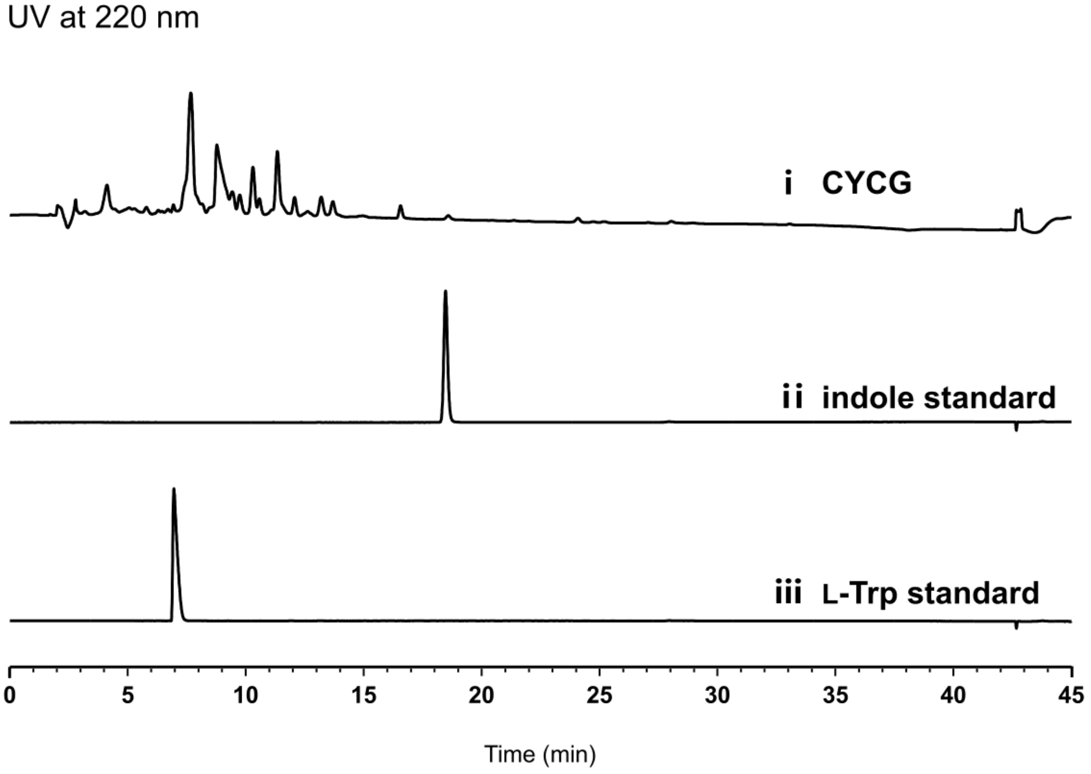

Figure S4. UV spectrum of meribisindole A (**4**)

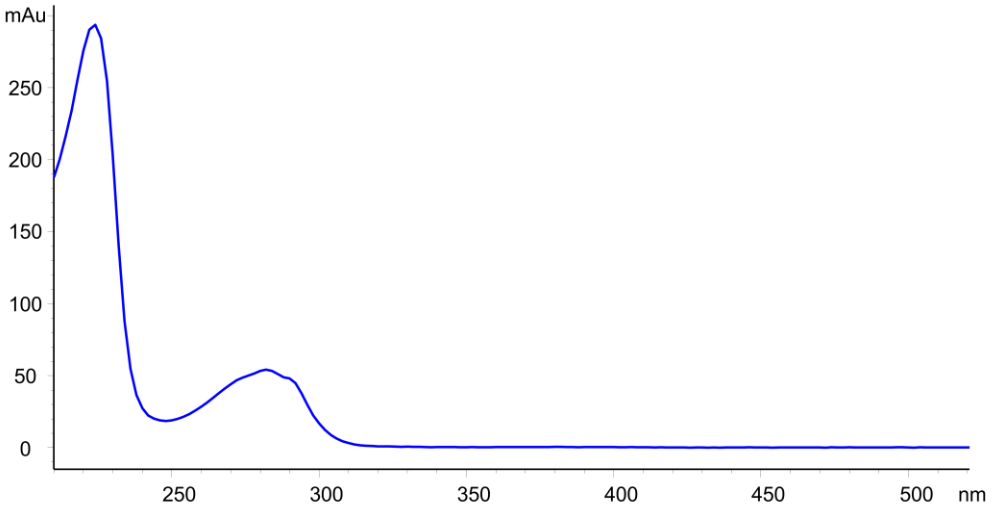

Figure S5. HR-ESI-MS spectrum of meribisindole A (**4**)

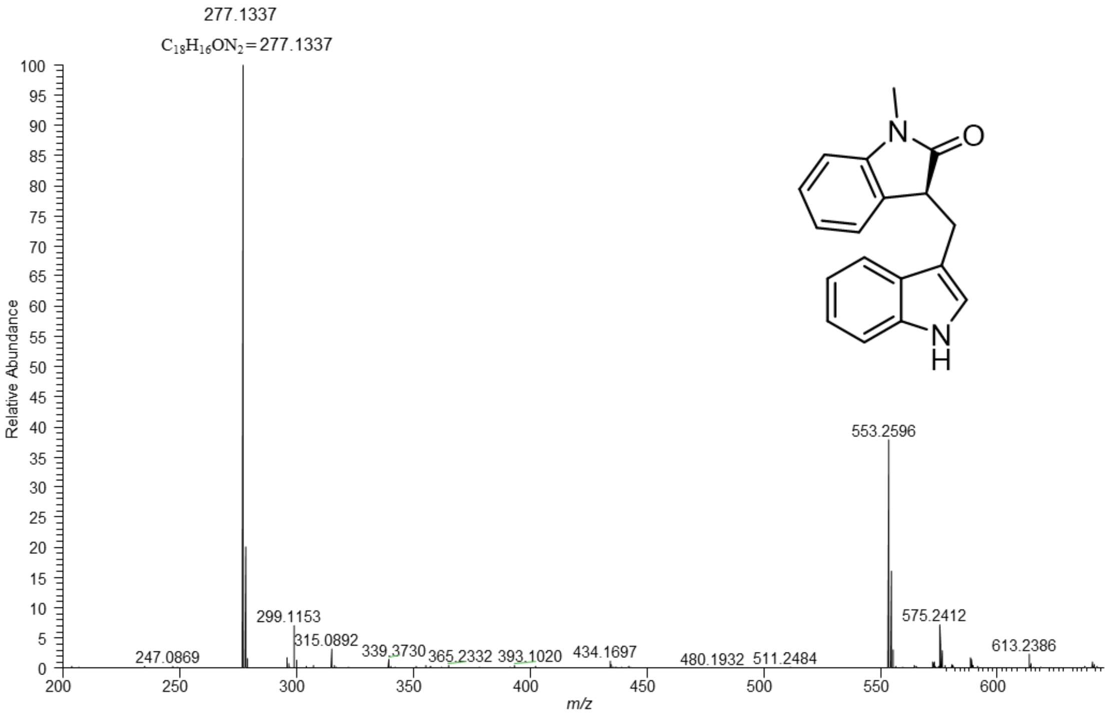

Figure S6. ^{1}H NMR spectrum of meribisindole A (**4**) in DMSO-d_6 (400 MHz)

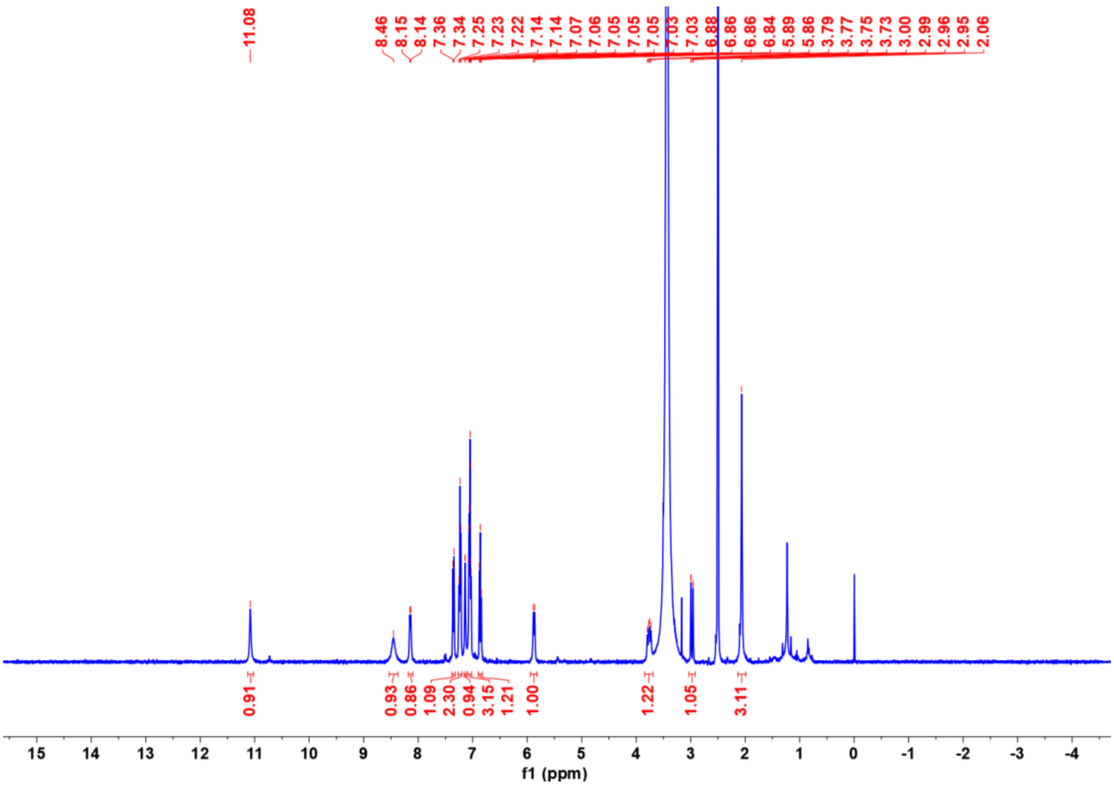

Figure S7. ^{13}C NMR spectrum of meribisindole A (**4**) in DMSO-d_6 (150 MHz)

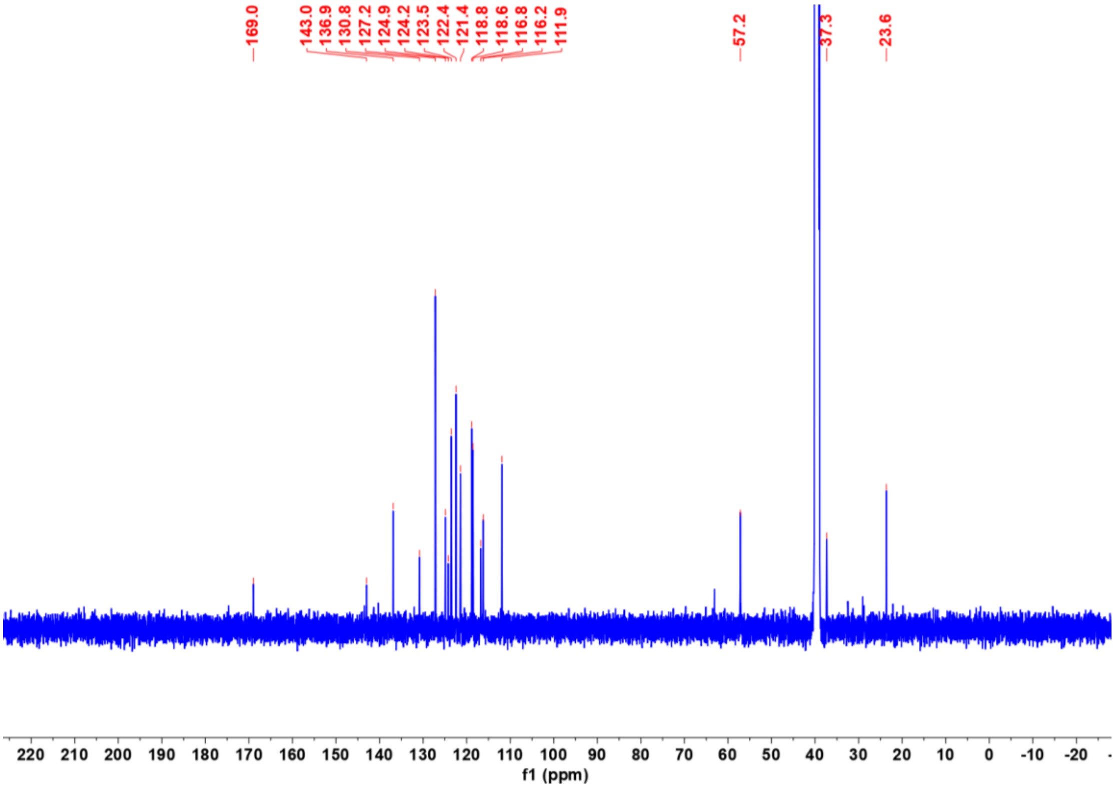

Figure S8. HSQC spectrum of meribisindole A (**4**) in DMSO-d_6

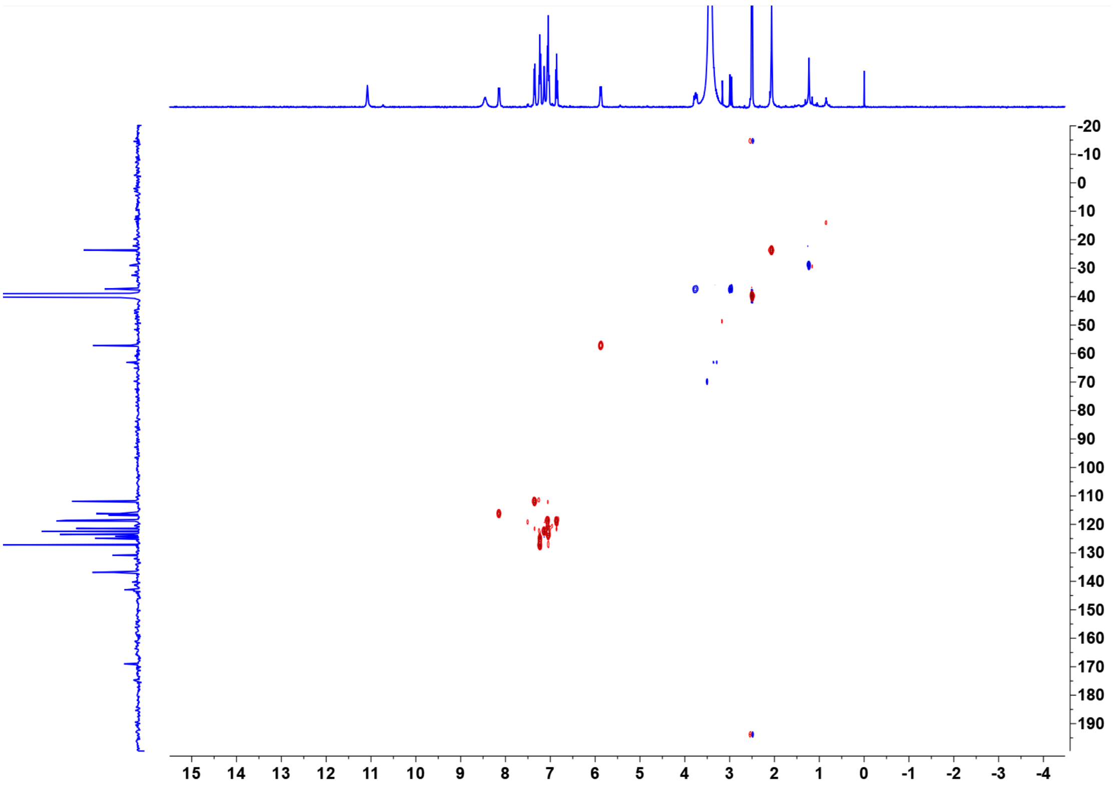

Figure S9. ^1H-^1H COSY spectrum of meribisindole A (**4**) in DMSO-d_6

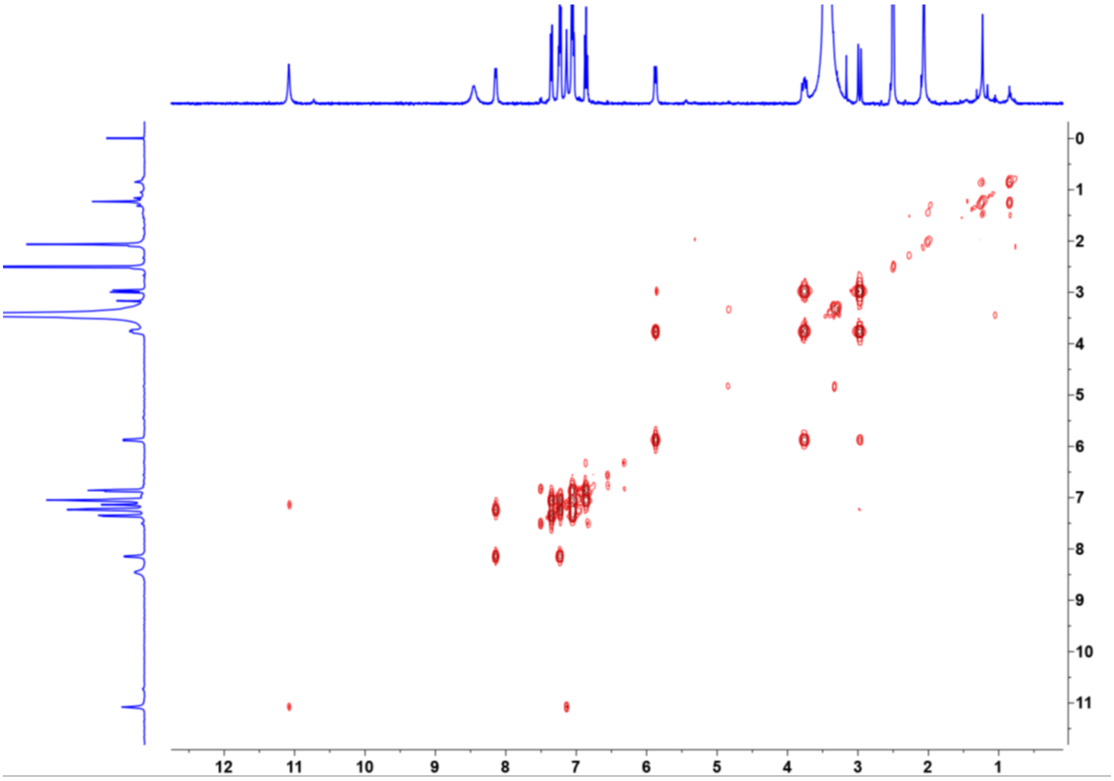

Figure S10. HMBC spectrum of meribisindole A (**4**) in DMSO-d_6

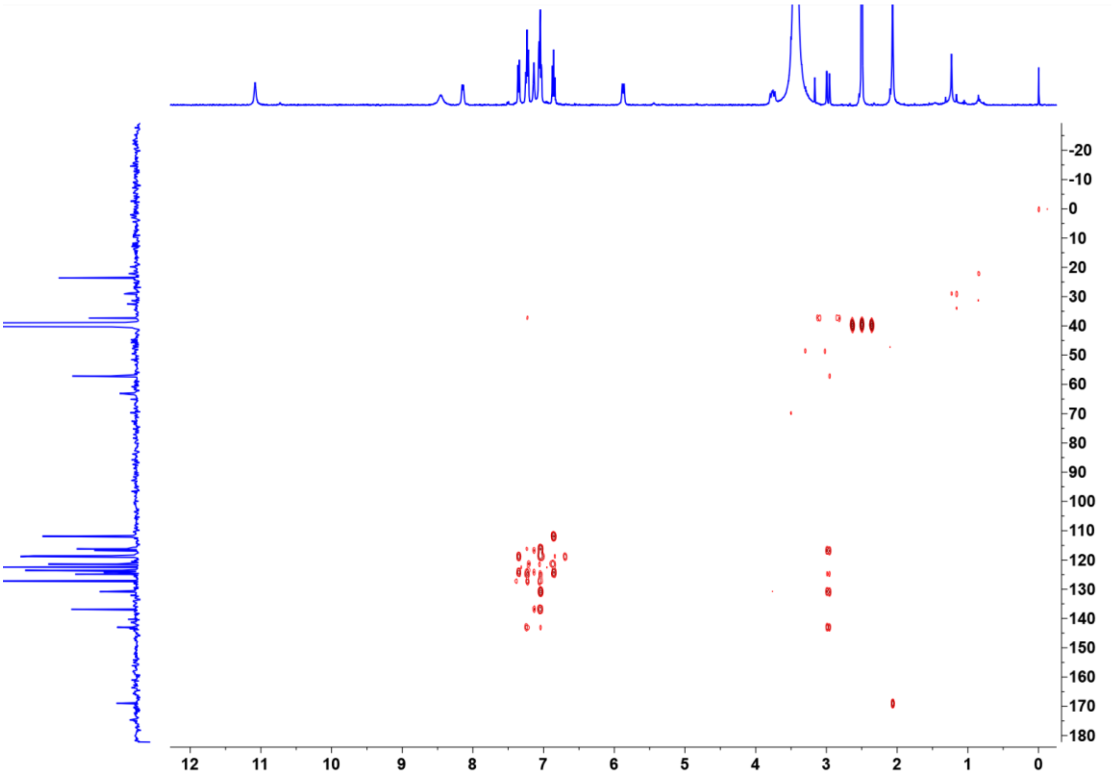

Figure S11. NOESY spectrum of meribisindole A (**4**) in DMSO-d_6

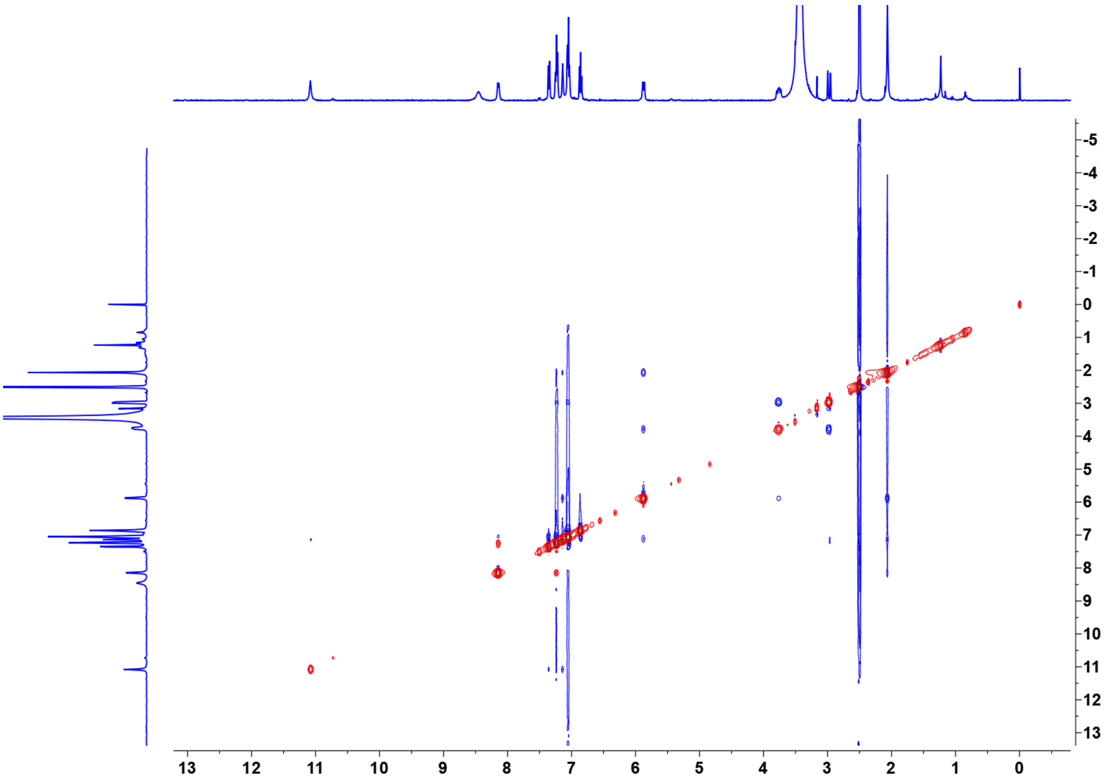

Figure S12. UV spectrum of meribisindole B (**5**)

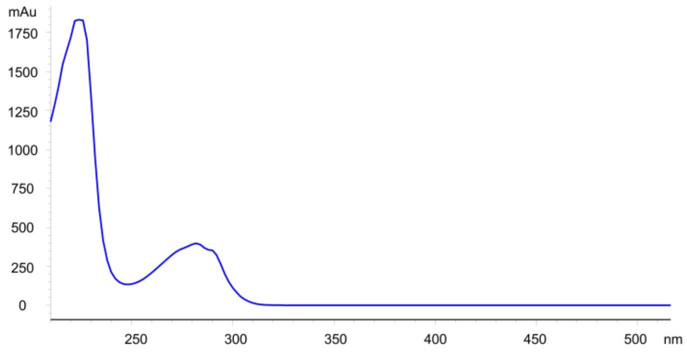

Figure S13. HR-ESI-MS spectrum of meribisindole B (**5**)

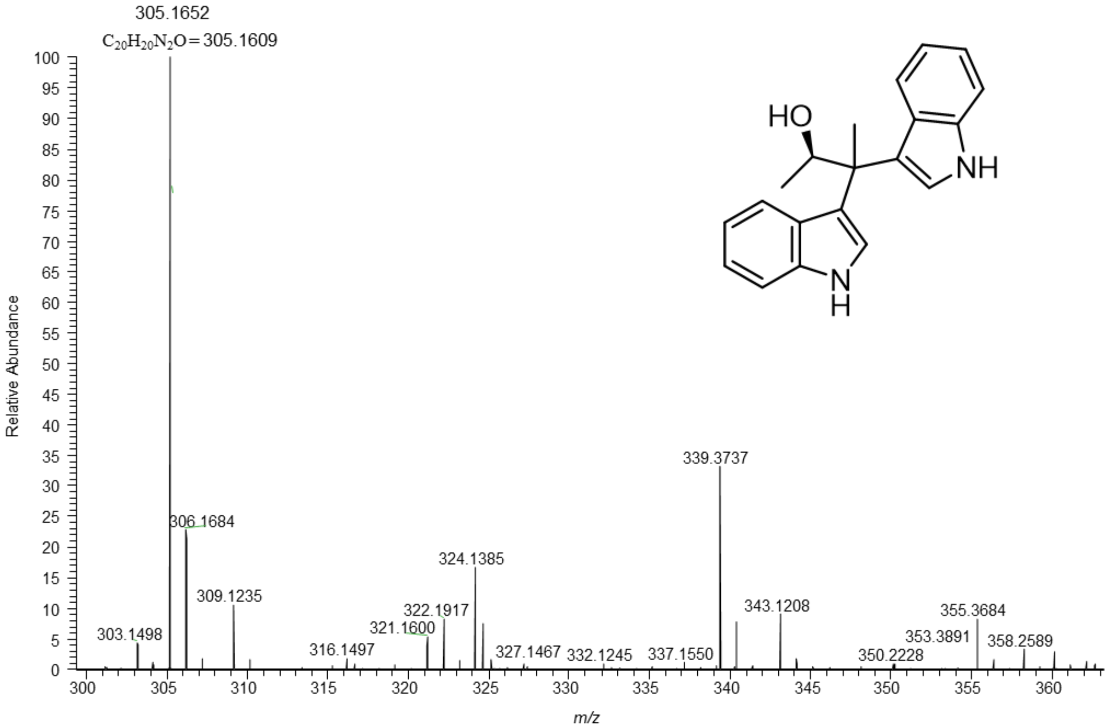

Figure S14. ^{1}H NMR spectrum of meribisindole B (**5**) in DMSO-d_6 (400 MHz)

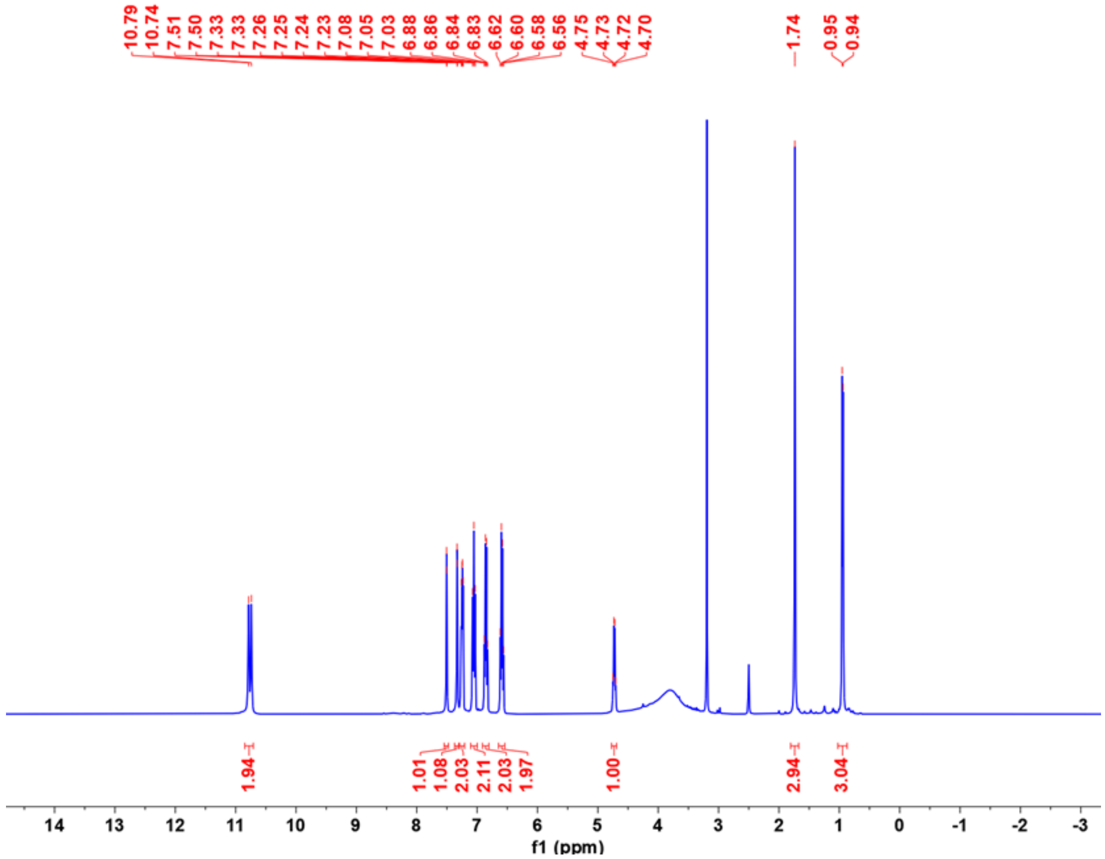

Figure S15. ^{13}C NMR spectrum of meribisindole B (**5**) in DMSO-d_6 (150 MHz)

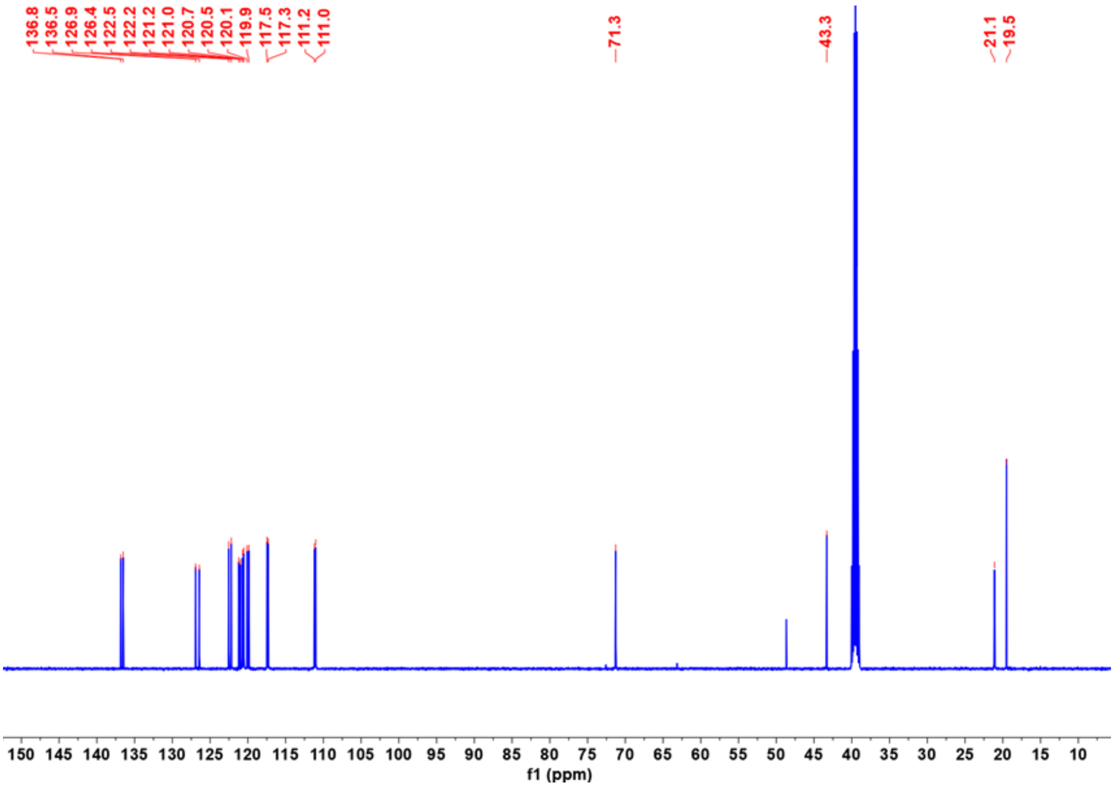

Figure S16. HSQC spectrum of meribisindole B (**5**) in DMSO-d_6

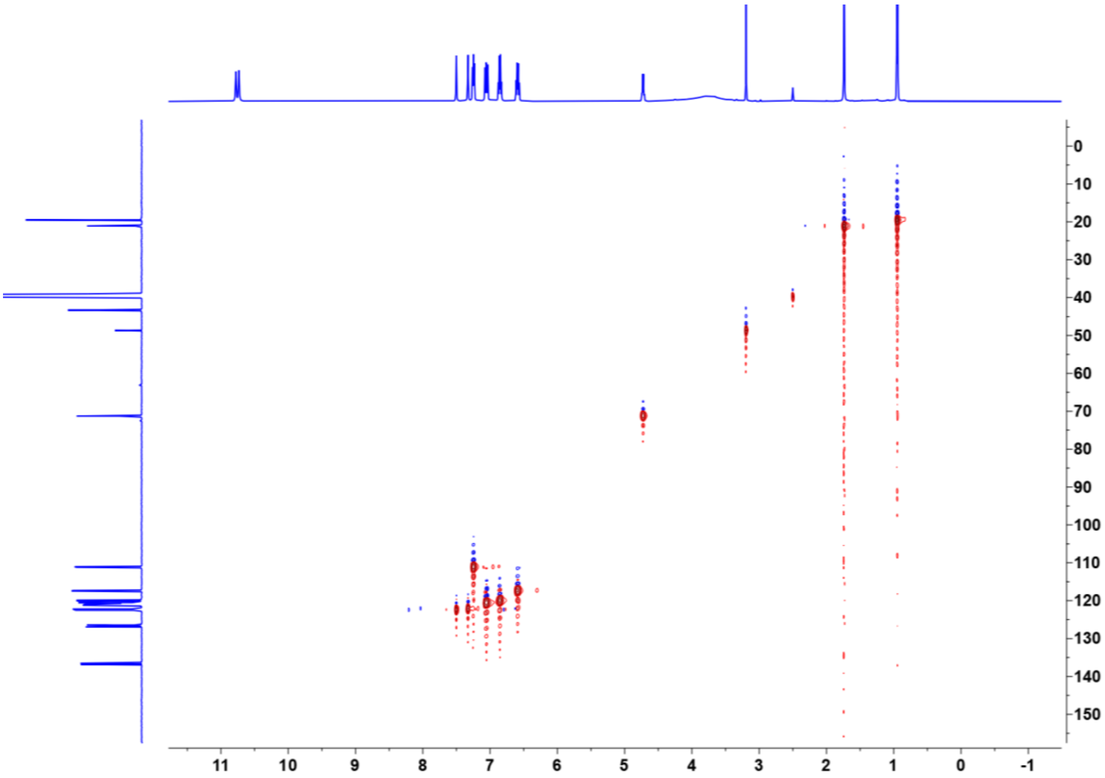

Figure S17. ^{1}H-^{1}H COSY spectrum of meribisindole B (**5**) in DMSO-d_6

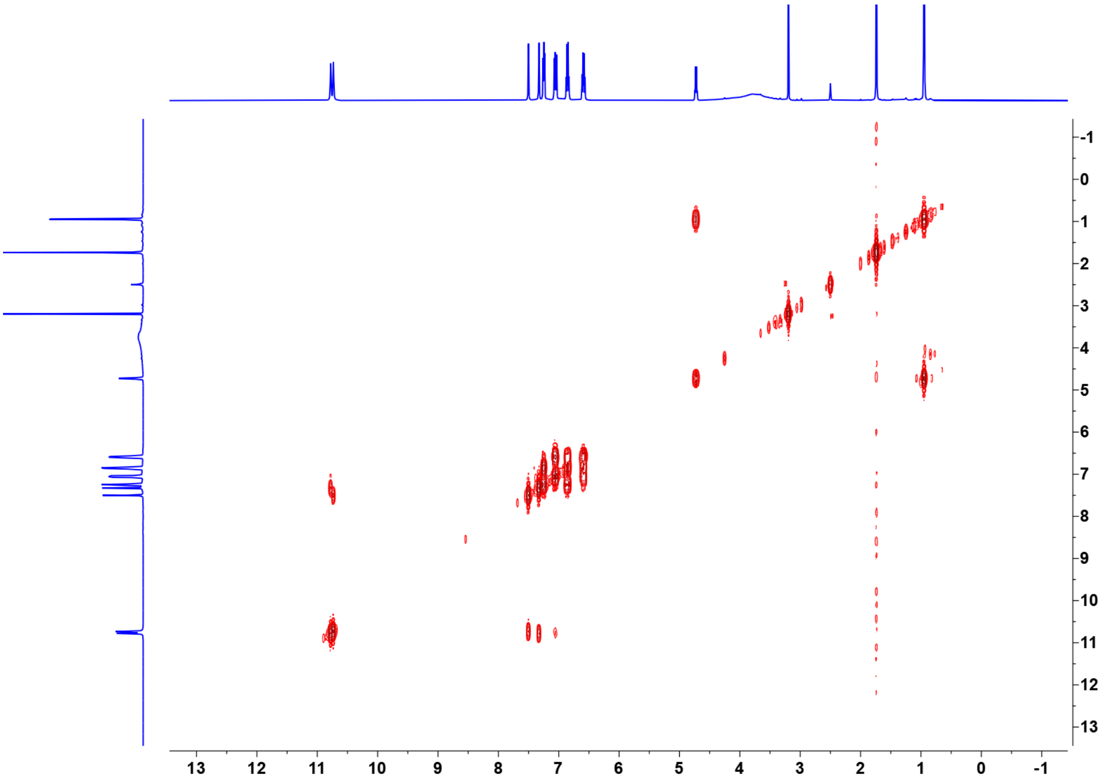

Figure S18. HMBC spectrum of meribisindole B (**5**) in DMSO-d_6

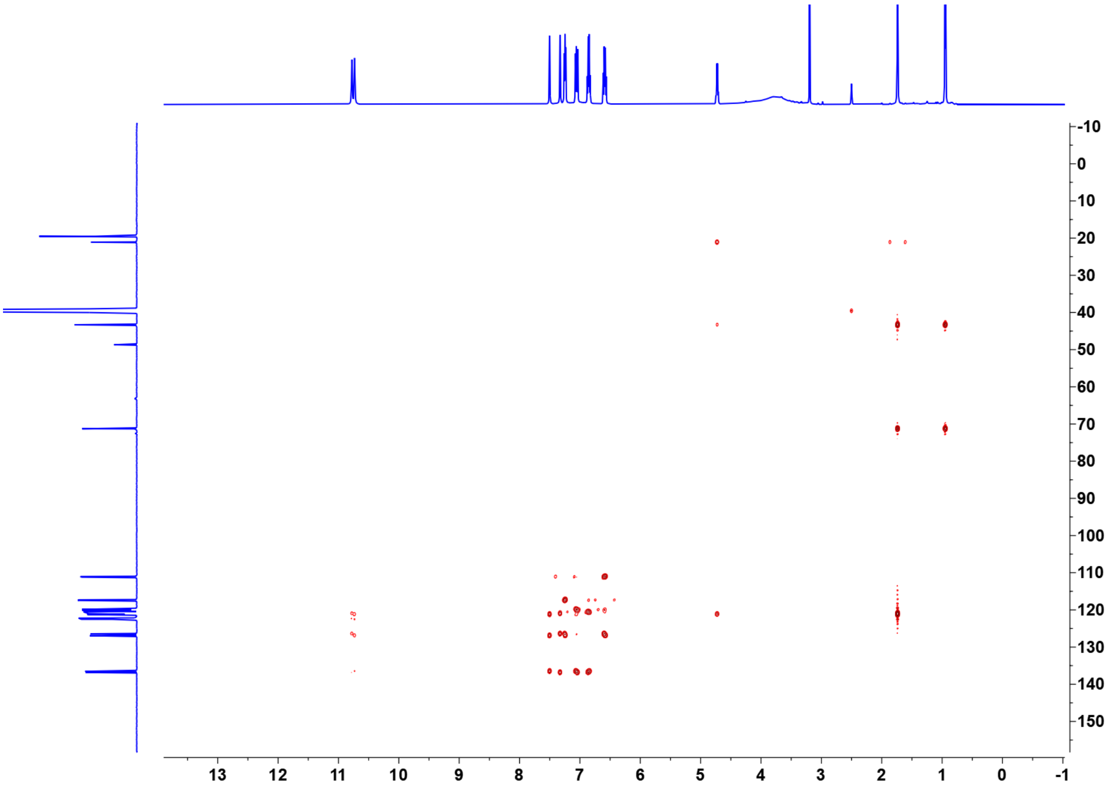

Figure S19. ^{1}H NMR spectrum of indole-3-carboxaldehyde (**1**) in DMSO-d_6 (600 MHz)

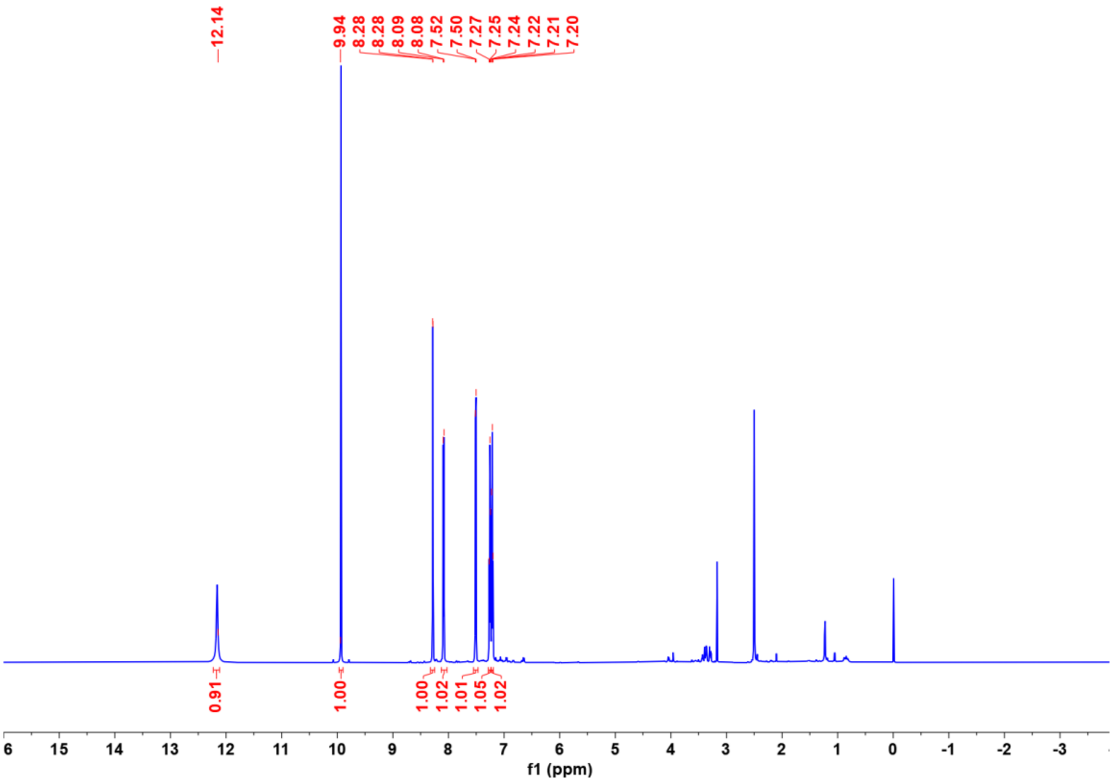

Figure S20. DEPTQ NMR spectrum of indole-3-carboxaldehyde (**1**) in DMSO-d_6 (150 MHz)

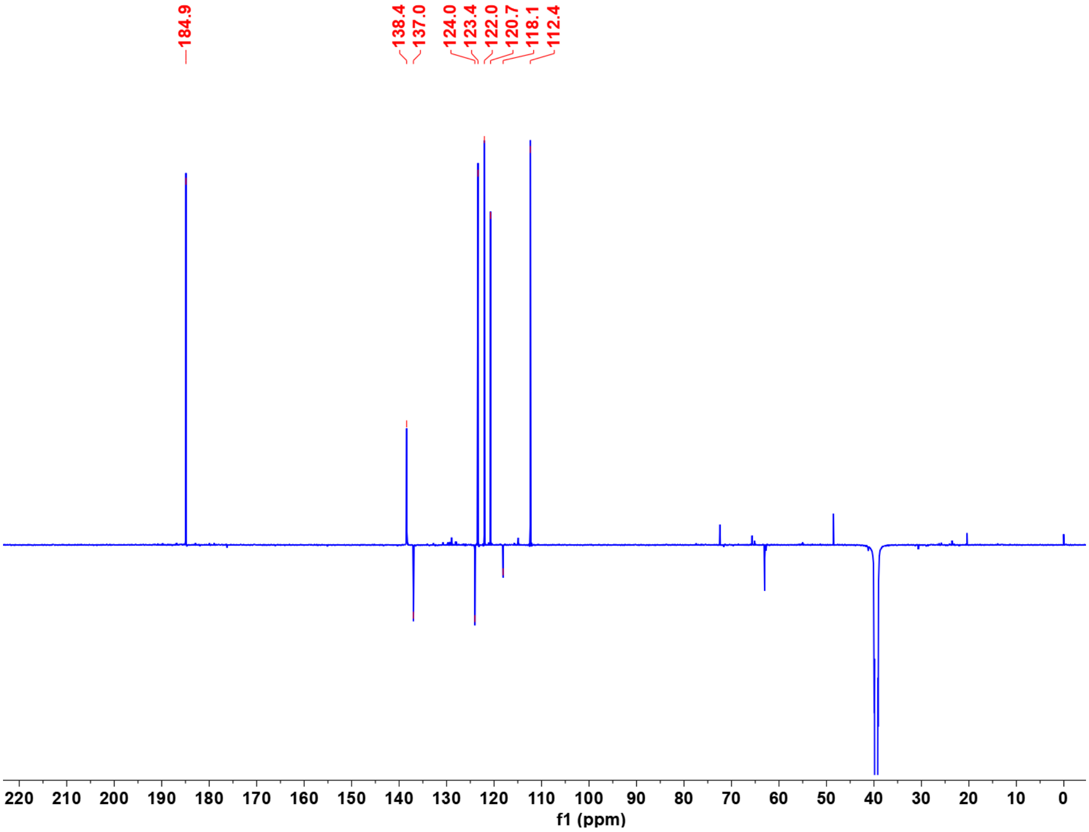

Figure S21. ^{1}H NMR spectrum of indole-3-acetic acid (**2**) in DMSO-d_6 (600 MHz)

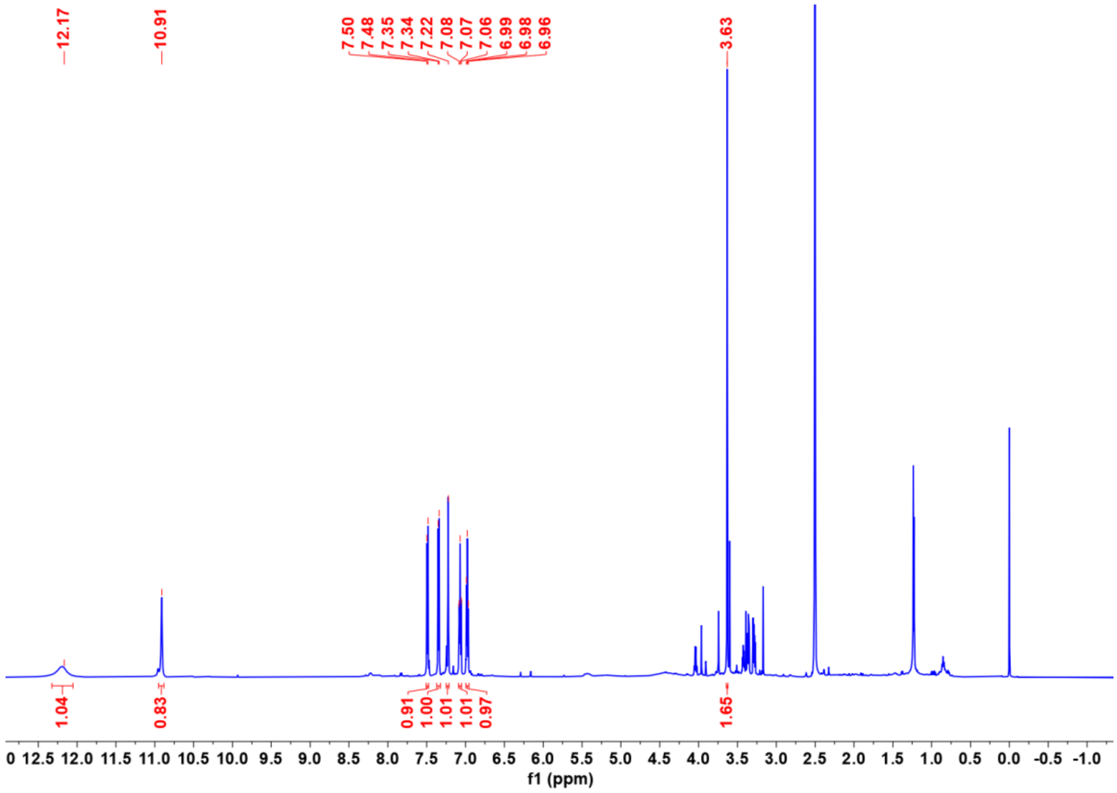

Figure S22. DEPTQ NMR spectrum of indole-3-acetic acid (**2**) in DMSO-*d*6 (150 MHz)

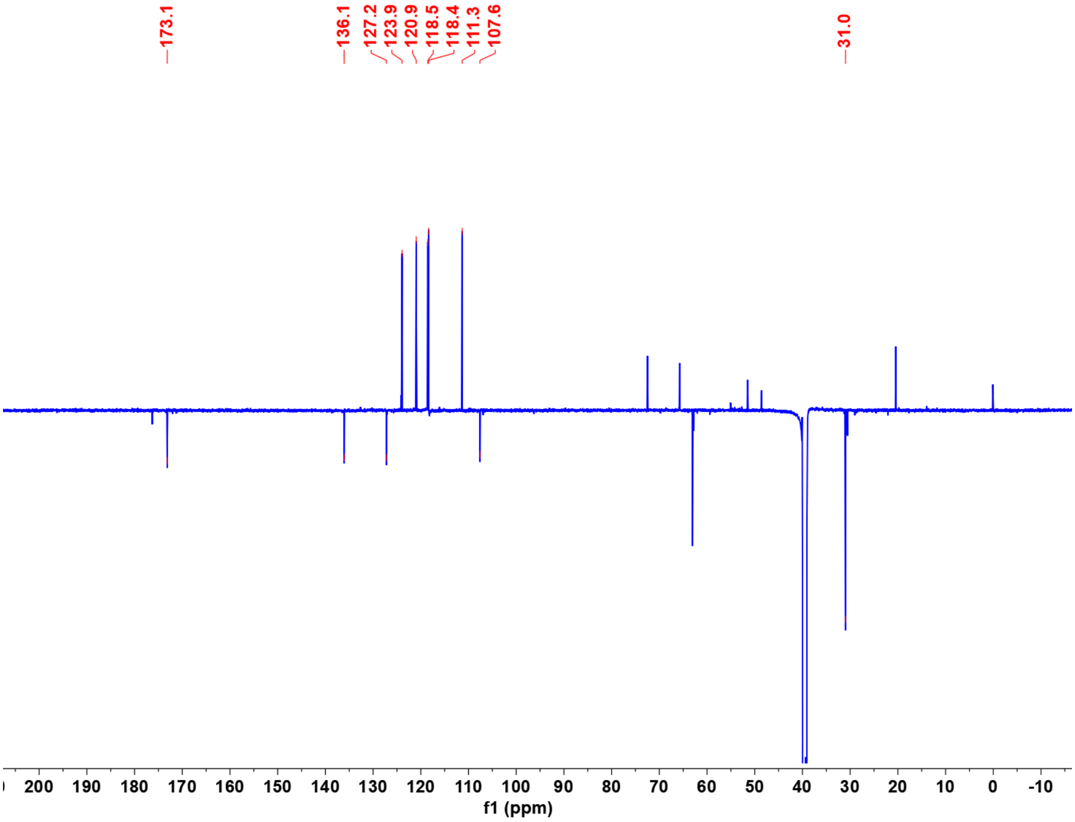

Figure S23. ^{1}H NMR spectrum of fusarindole B (**3**) in DMSO-d_6 (400 MHz)

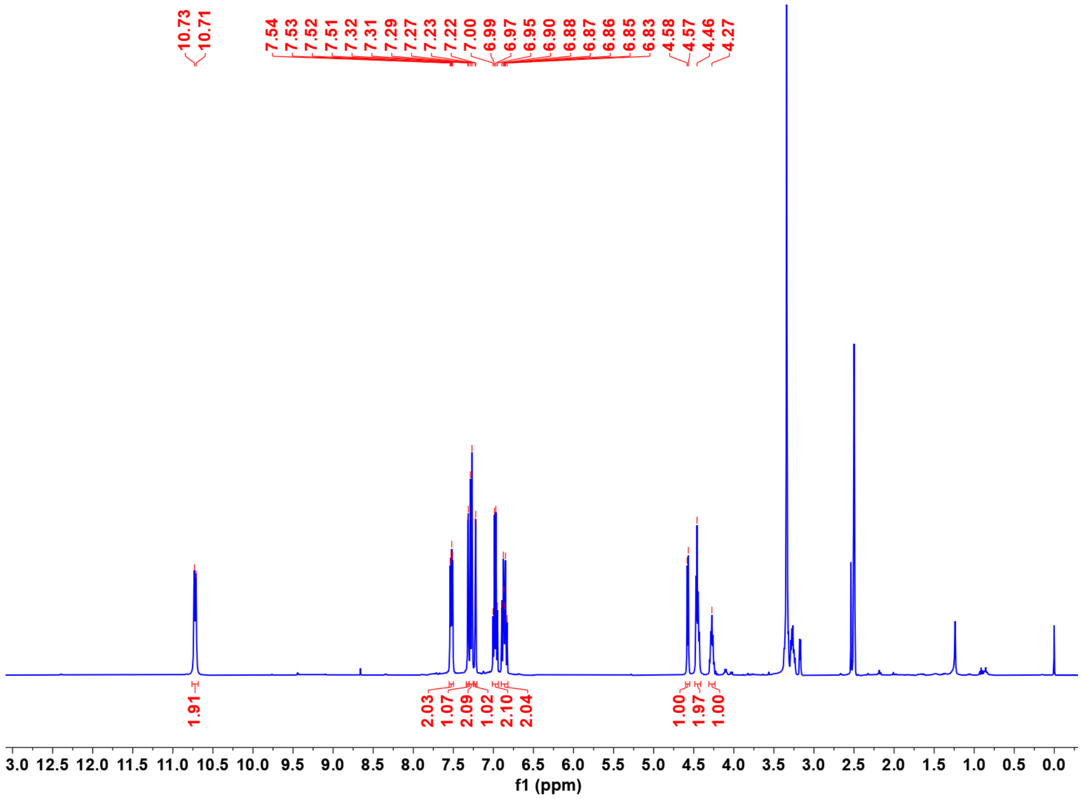

Figure S24. ^{13}C NMR spectrum of fusarindole B (**3**) in DMSO-d_6 (150 MHz)

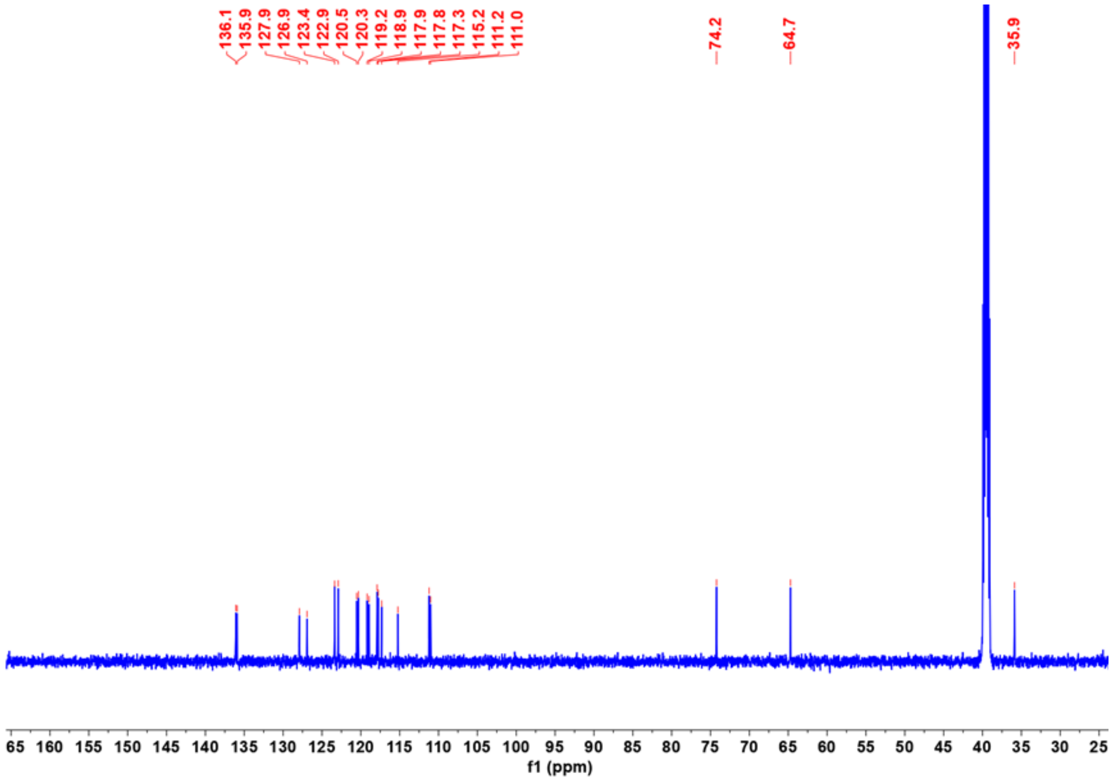

Figure S25. ^{1}H NMR spectrum of compounds 3,3′-biindole (**6**) in DMSO-d_6 (400 MHz)

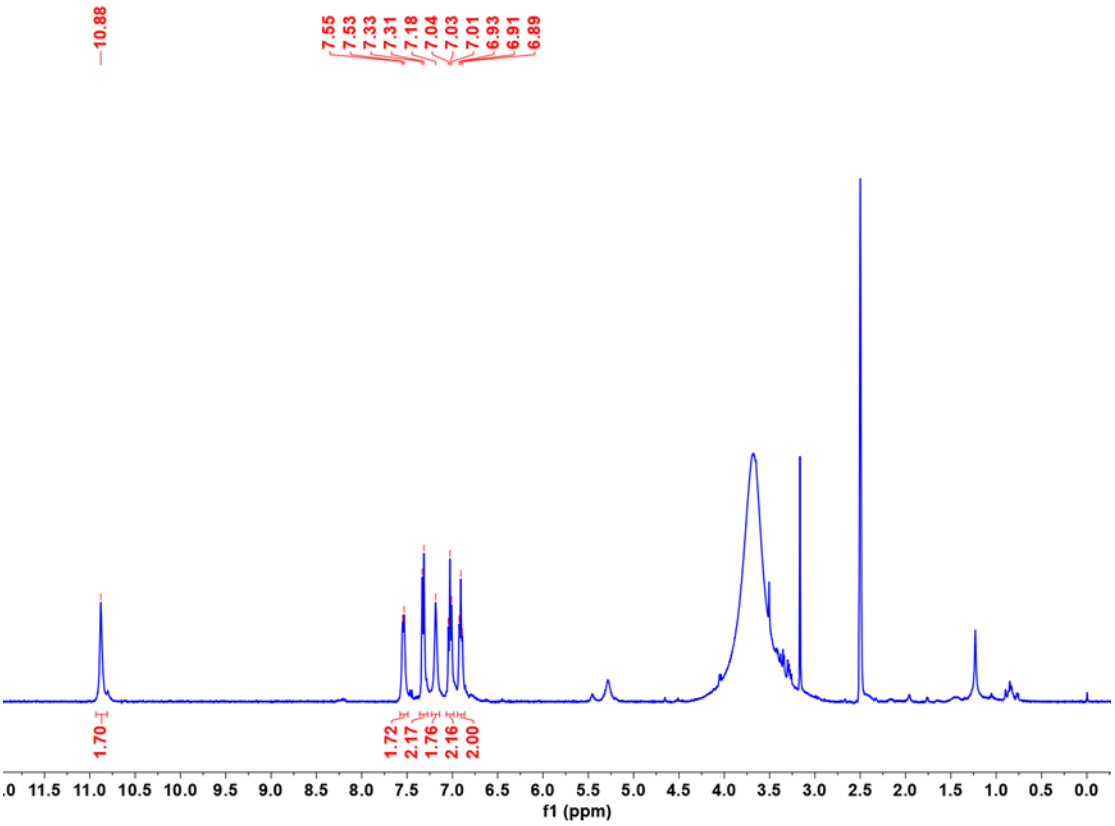

Figure S26. ^{13}C NMR spectrum of compounds 3,3′-biindole (**6**) in DMSO-d_6 (150 MHz)

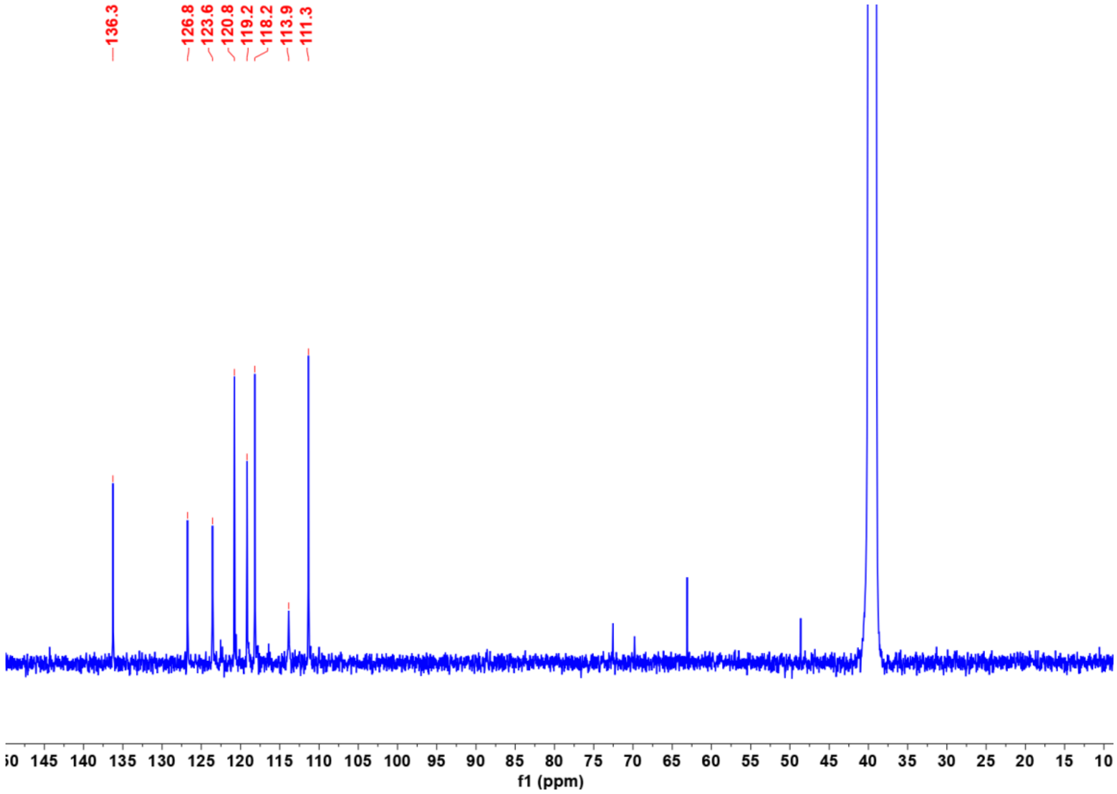

Figure S27. ^{1}H NMR spectrum of 3,3′-diindolylmethane (**7**) in DMSO-d_6 (600 MHz)

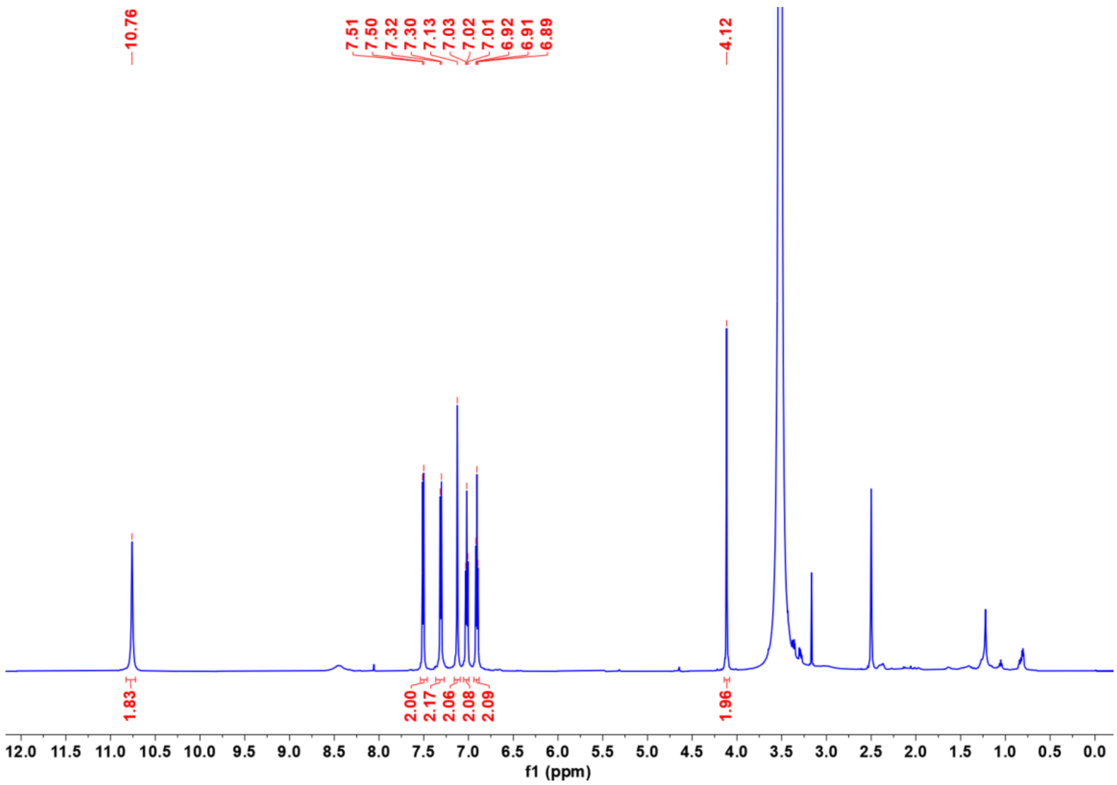

Figure S28. DEPTQ NMR spectrum of 3,3′-diindolylmethane (**11**) in DMSO-*d*6 (150 MHz)

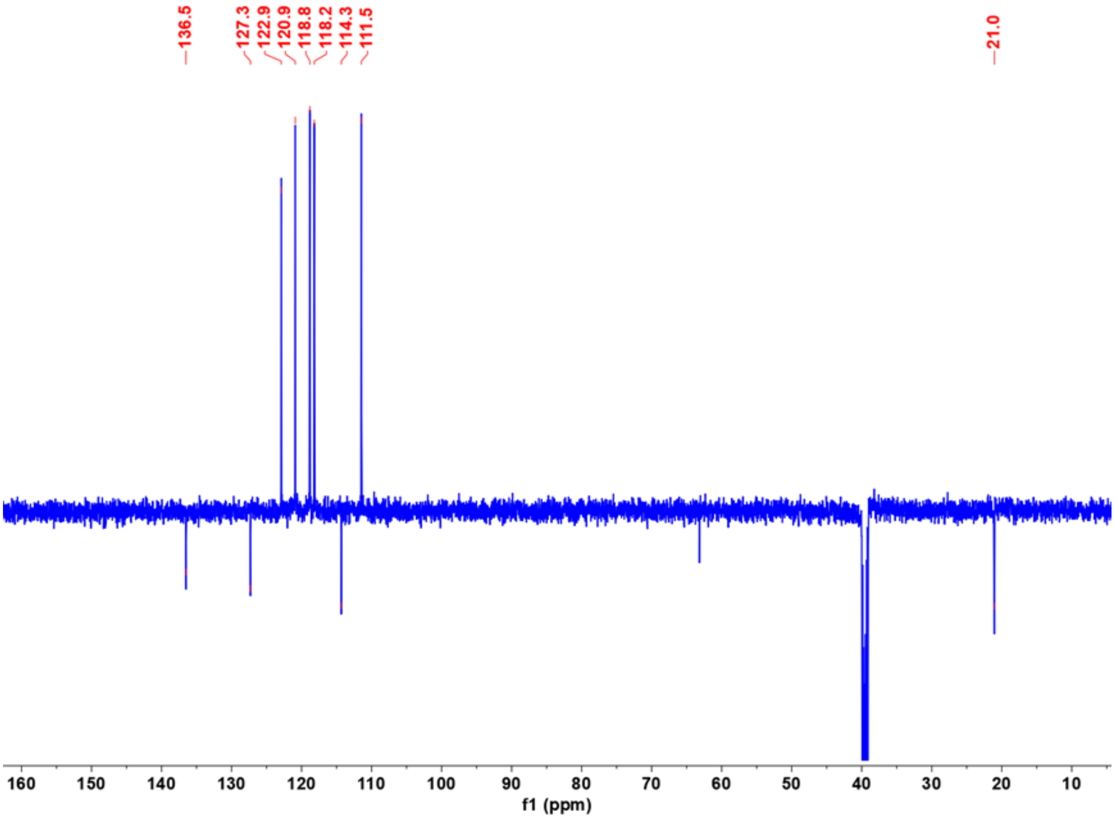

Figure S29. ^{1}H NMR spectrum of vibrindole A (**8**) in DMSO-d_6 (600 MHz)

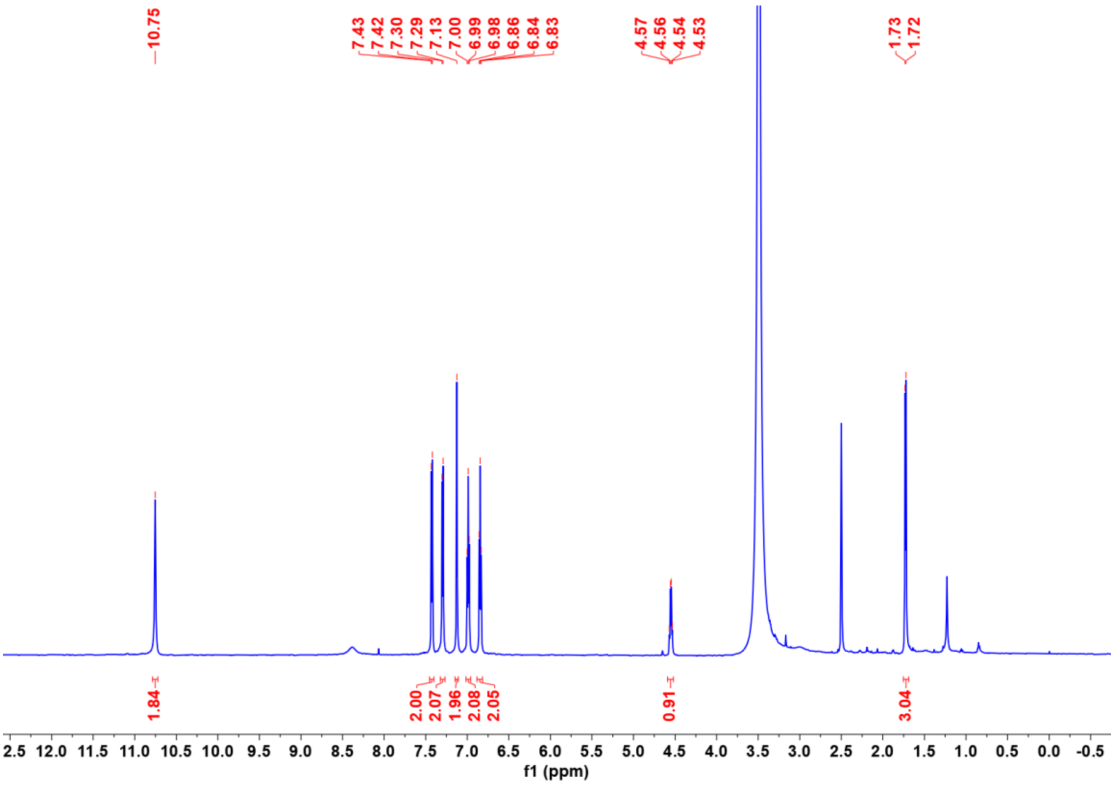

Figure S30. DEPTQ NMR spectrum of vibrindole A (**8**) in DMSO-d_6 (150 MHz)

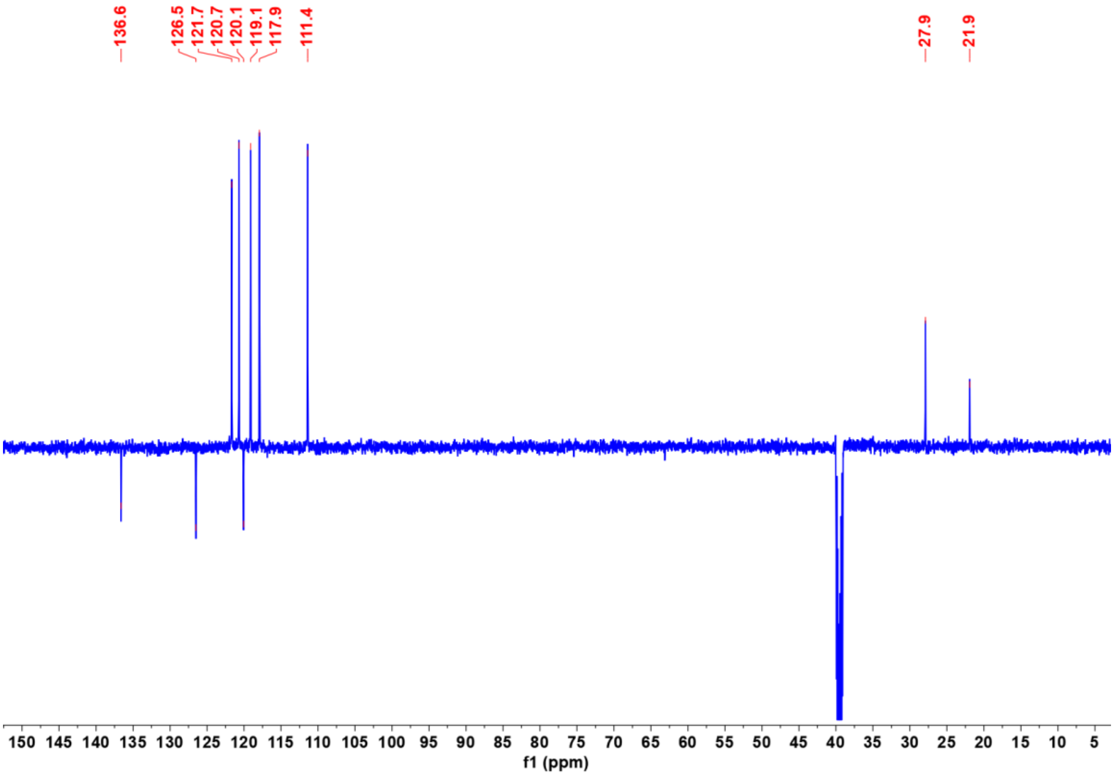

Figure S31. ^{1}H NMR spectrum of 3,3′-(1-methylethylidene)-bis-[1*H*-indole] (**9**) in DMSO-d_6 (400 MHz)

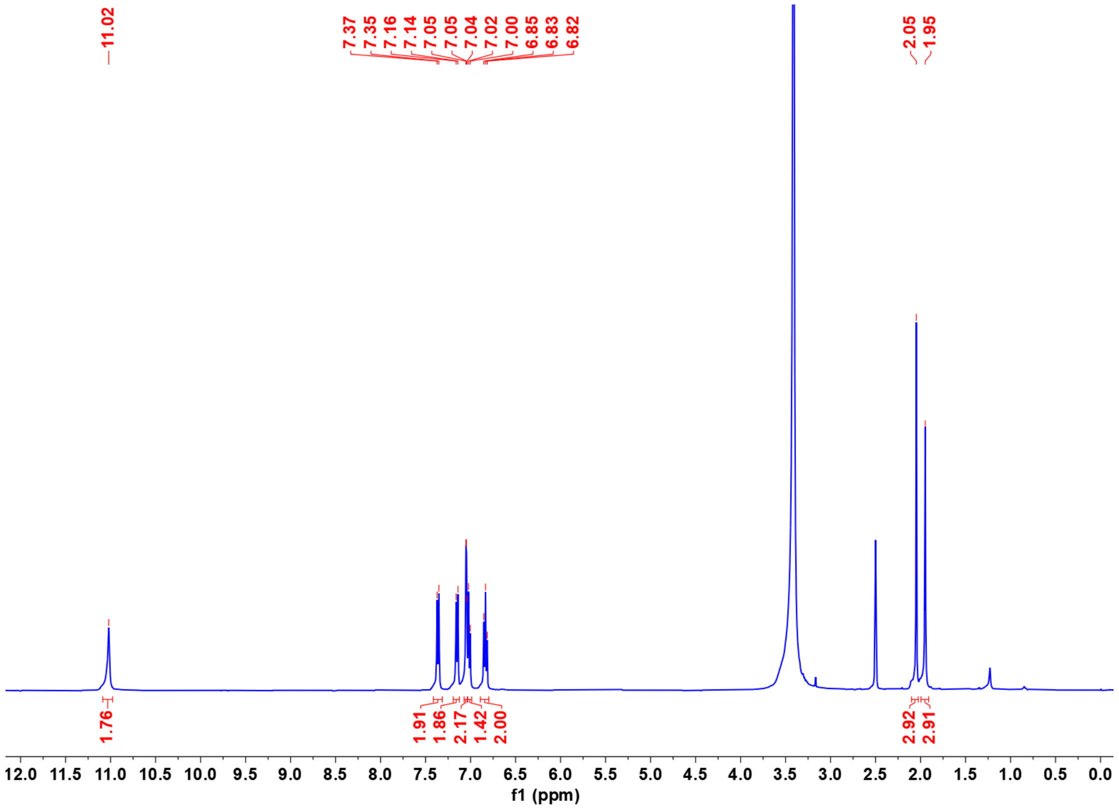

Figure S32. DEPTQ NMR spectrum of 3,3′-(1-methylethylidene)-bis-[1*H*-indole] (**9**) in DMSO-*d*6 (150 MHz)

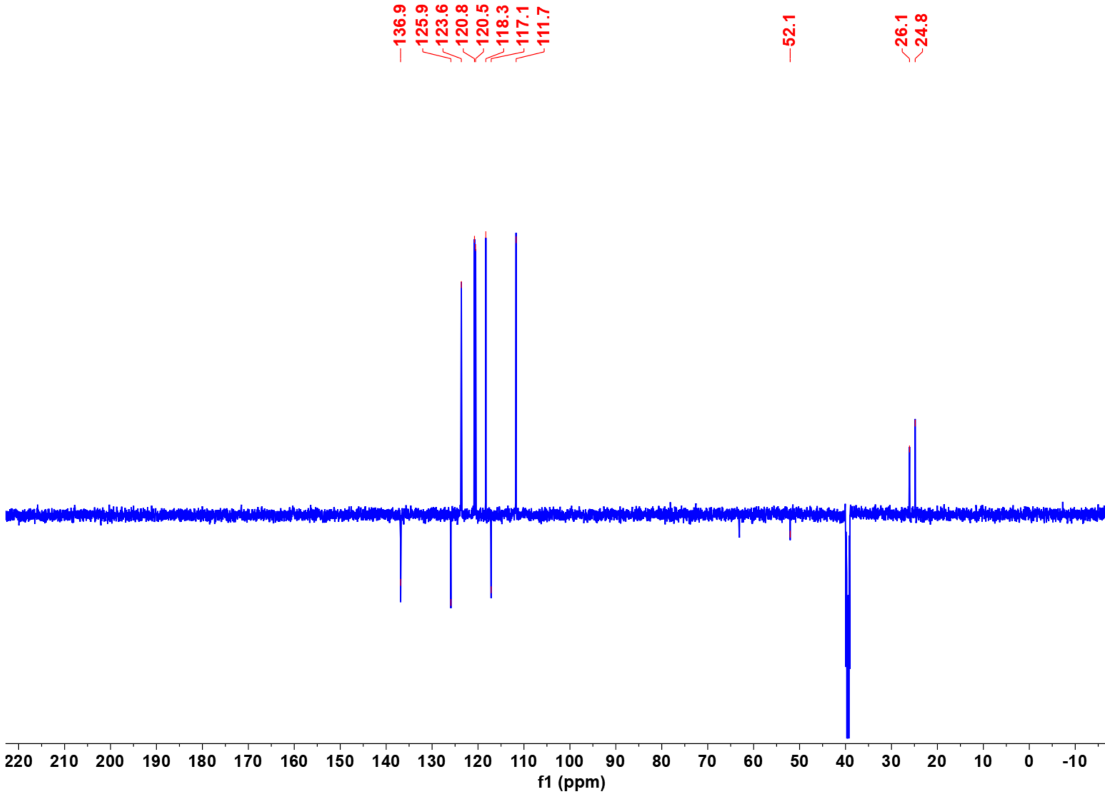

Figure S33. ^{1}H NMR spectrum of 3,3′,3″-mthanetriyltris-1*H*-indole (**10**) in DMSO-d_6 (600 MHz)

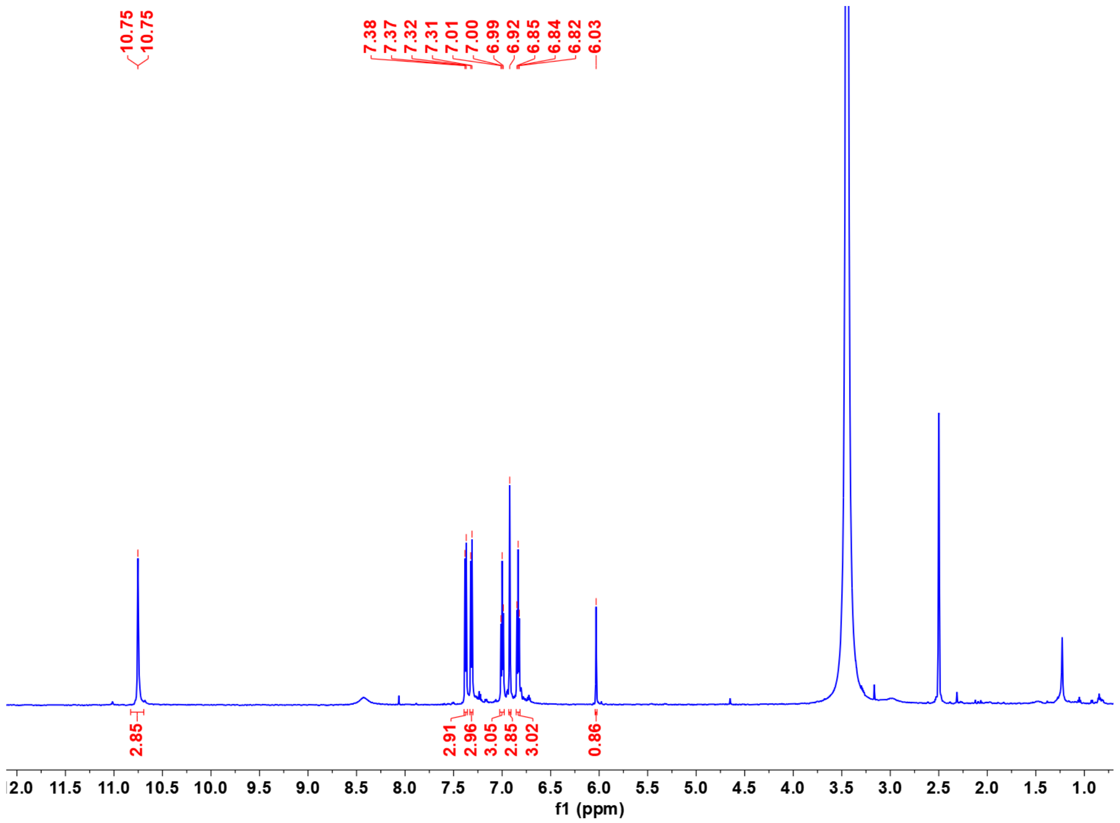

Figure S34. DEPTQ NMR spectrum of 3,3′,3′′-mthanetriyltris-1*H*-indole (**10**) in DMSO-d_6 (150 MHz)

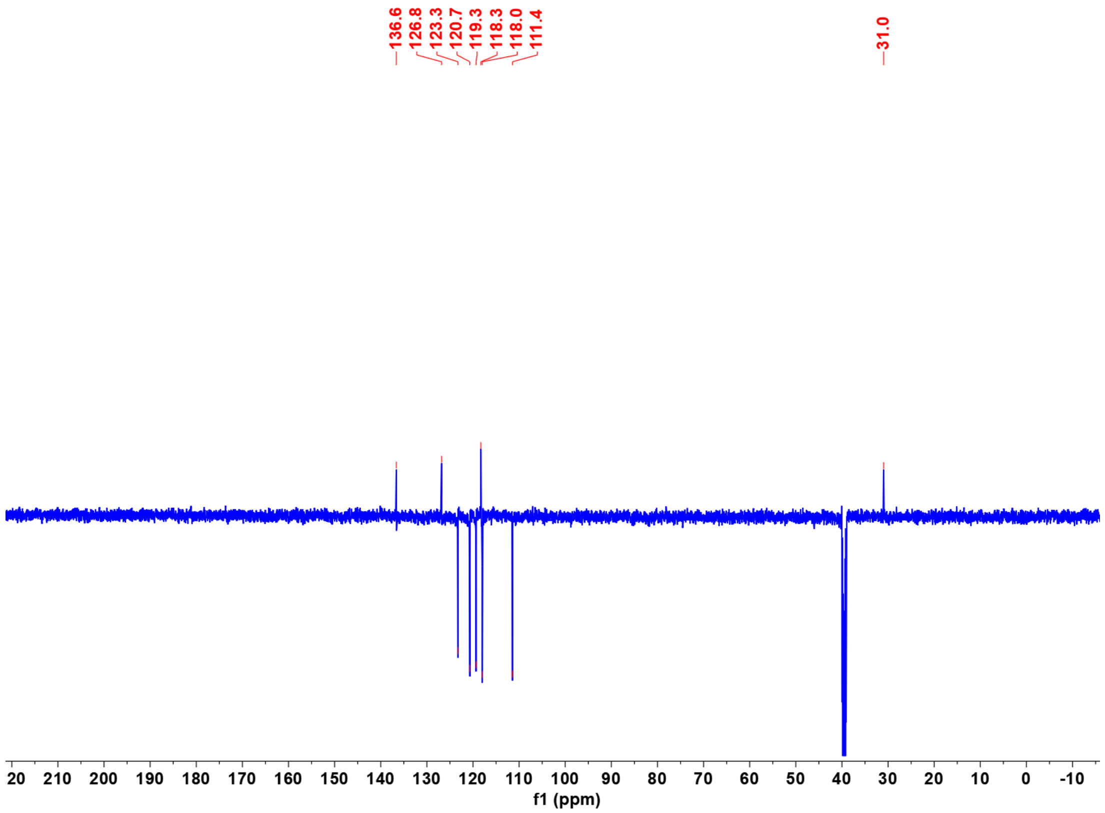

Figure S35. ^{1}H NMR spectrum of 3,3-bis(1*H*-indol-3-yl)-1*H*-indol-2-one (**11**) in DMSO-d_6 (400 MHz)

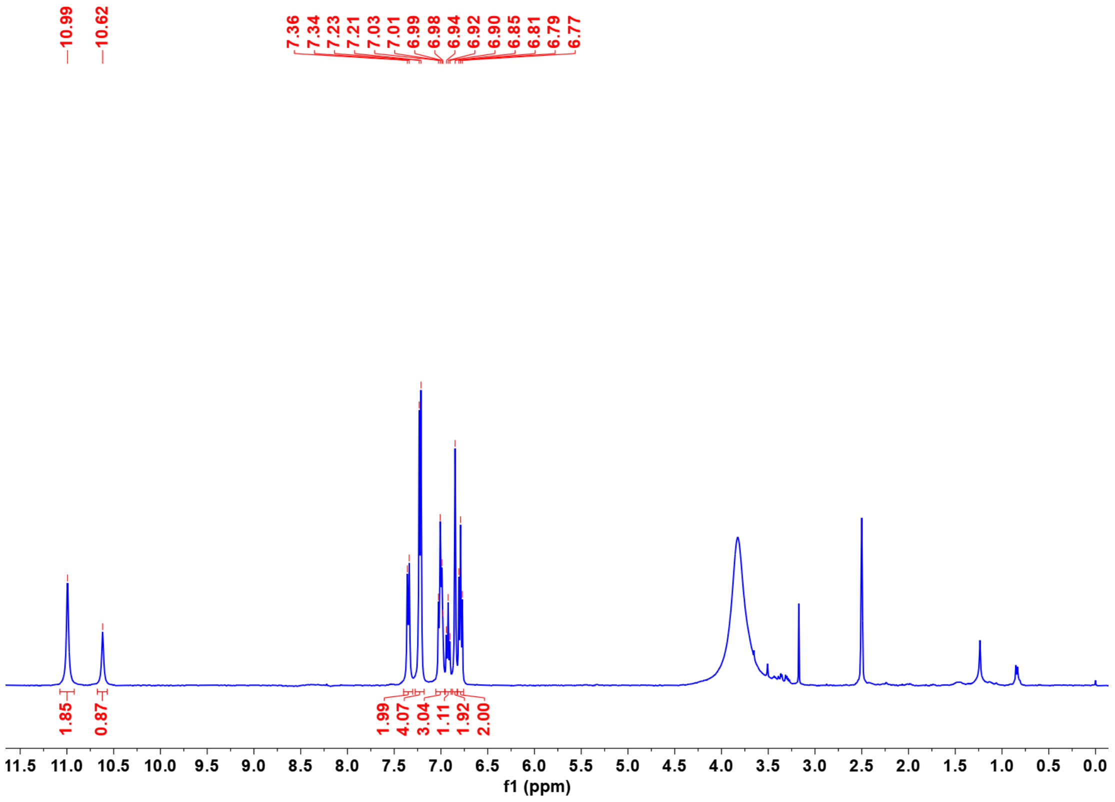

Figure S36. ^{13}C NMR spectrum of 3,3-bis(1*H*-indol-3-yl)-1*H*-indol-2-one (**11**) in DMSO-d_6 (150 MHz)

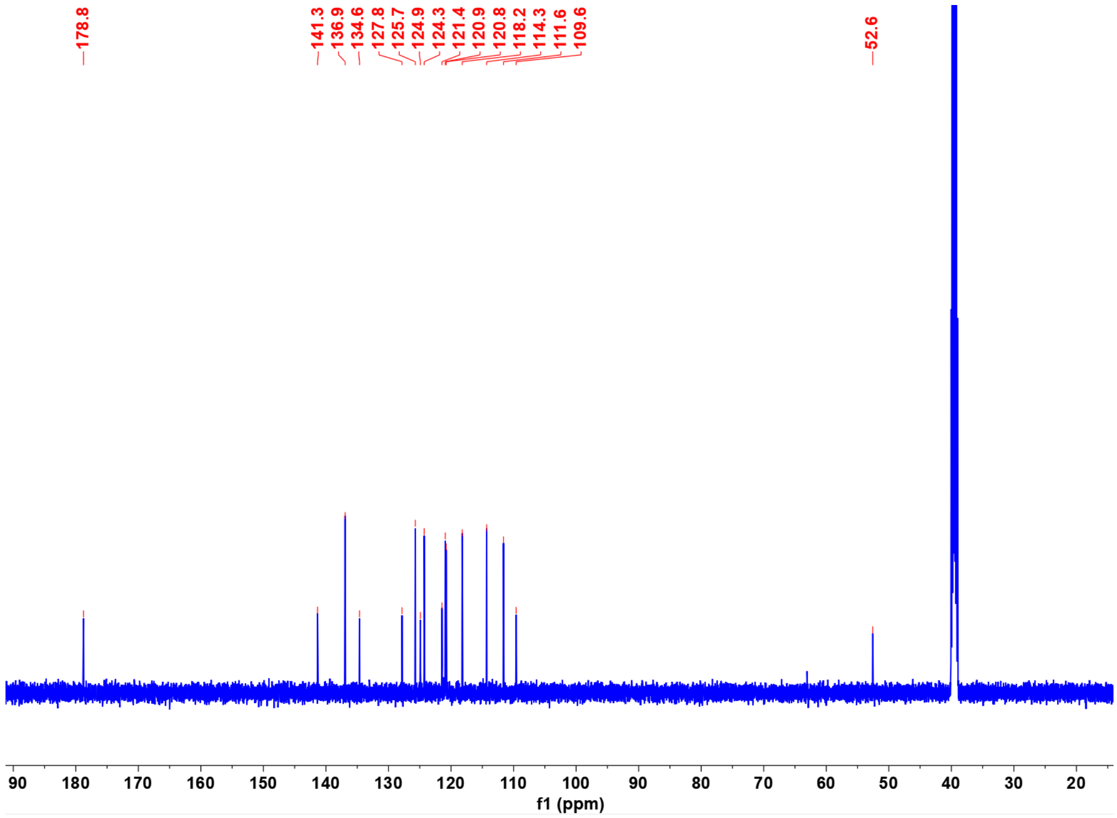

Figure S37. ^{1}H NMR spectrum of 2,2-bis(1*H*-indol-3-yl)indolin-3-one (**12**) in DMSO-d_6 (400 MHz)

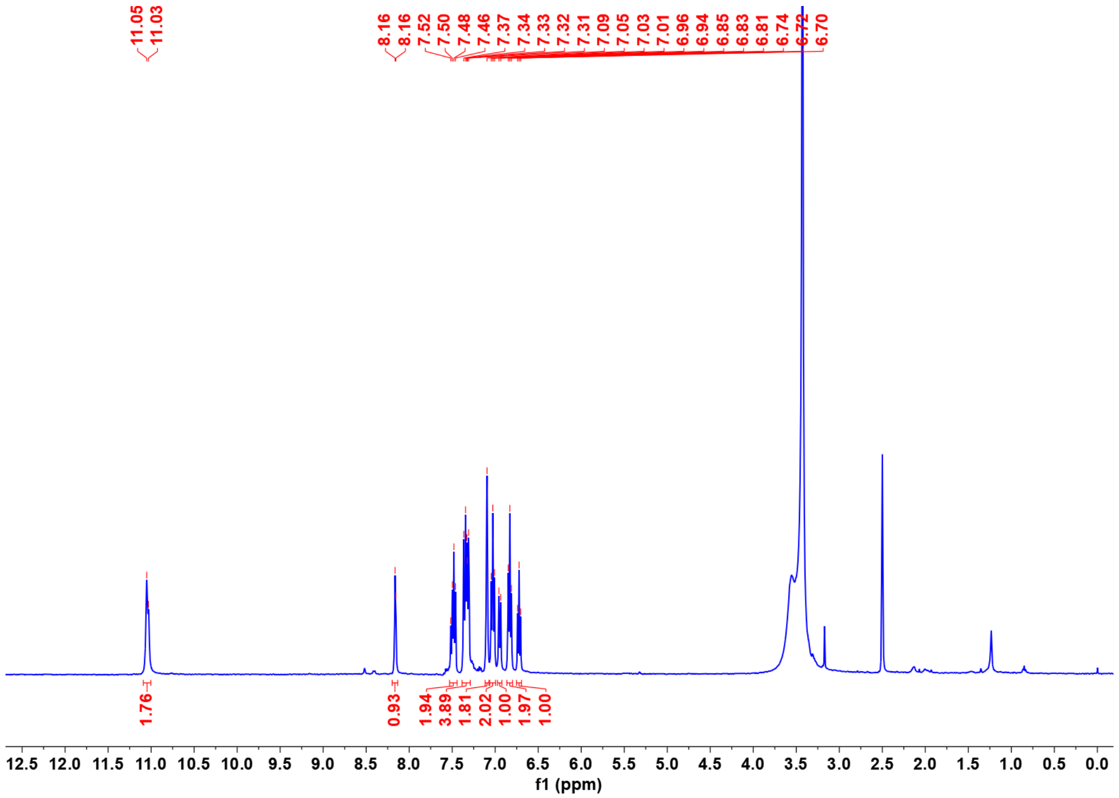

Figure S38. ^{13}C NMR spectrum of 2,2-bis(1*H*-indol-3-yl)indolin-3-one (**12**) in DMSO-d_6 (150 MHz)

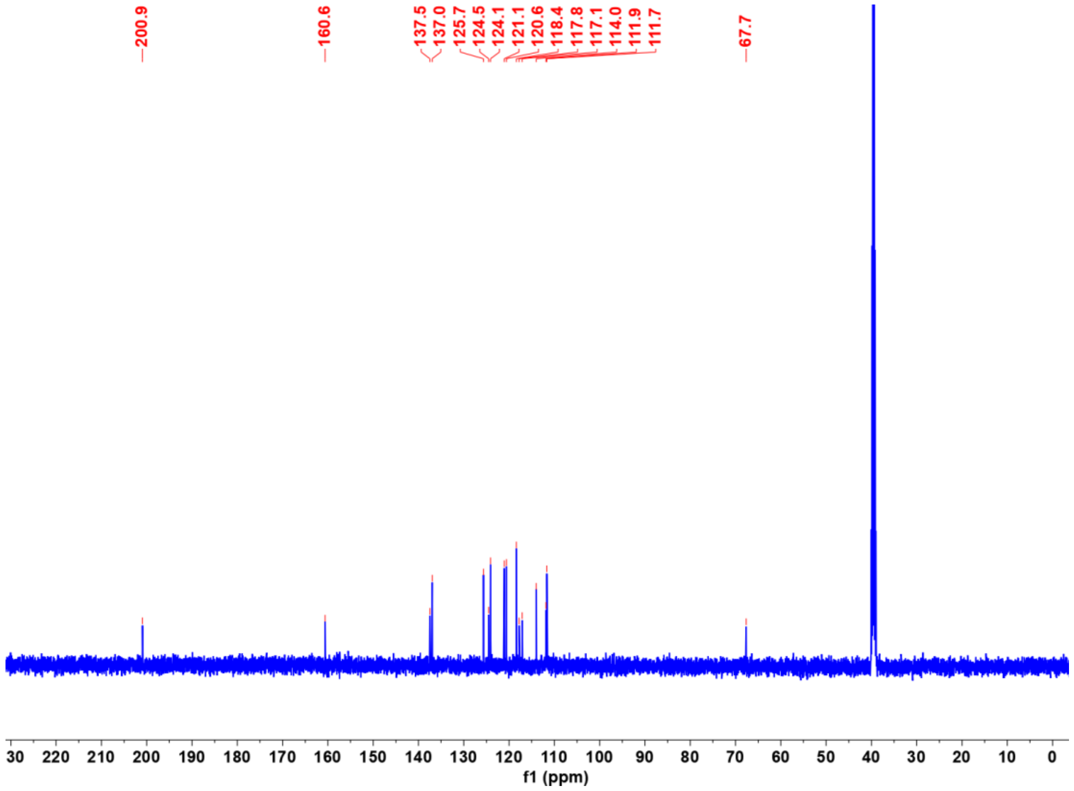

Figure S39. ^{1}H NMR spectrum of metagenetriindole A (**13**) in DMSO-d_6 (400 MHz)

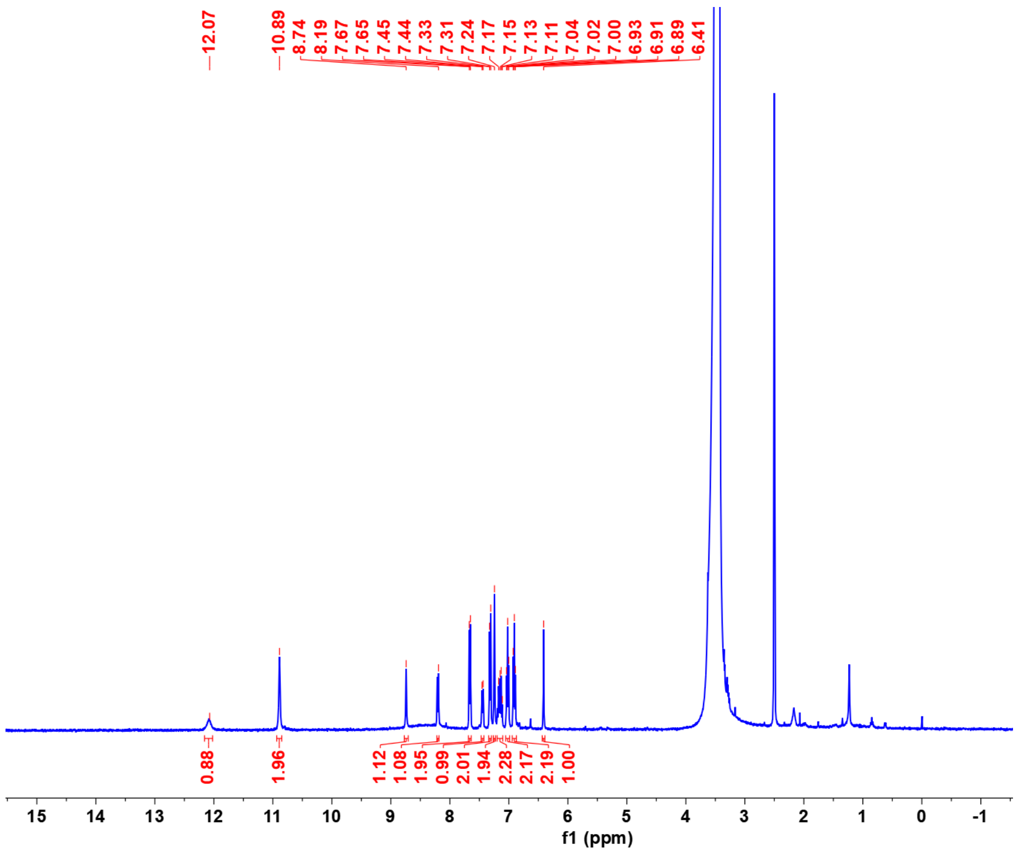

Figure S40. DEPTQ NMR spectrum of metagenetriindole A (**13**) in DMSO-d_6 (150 MHz)

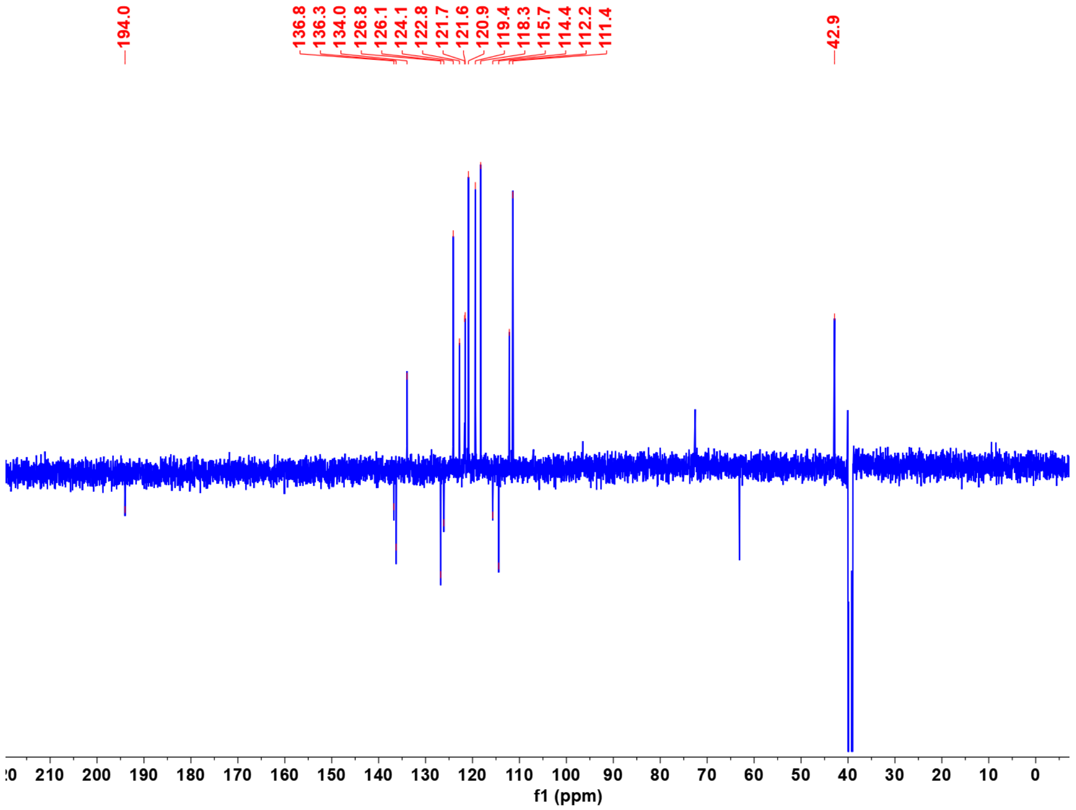

Figure S41. ^{1}H NMR spectrum of metagenetriindole A (**14**) in DMSO-d_6 (400 MHz)

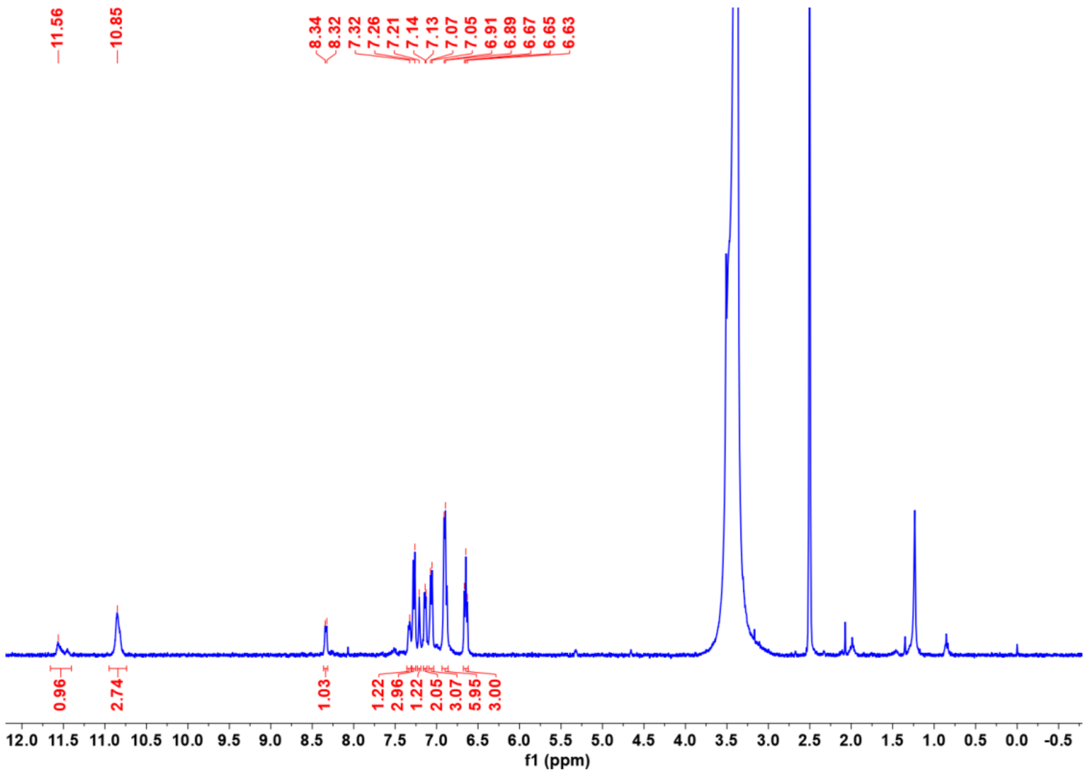

Figure S42. ^{13}C NMR spectrum of metagenetriindole A (**14**) in DMSO-d_6 (150 MHz)

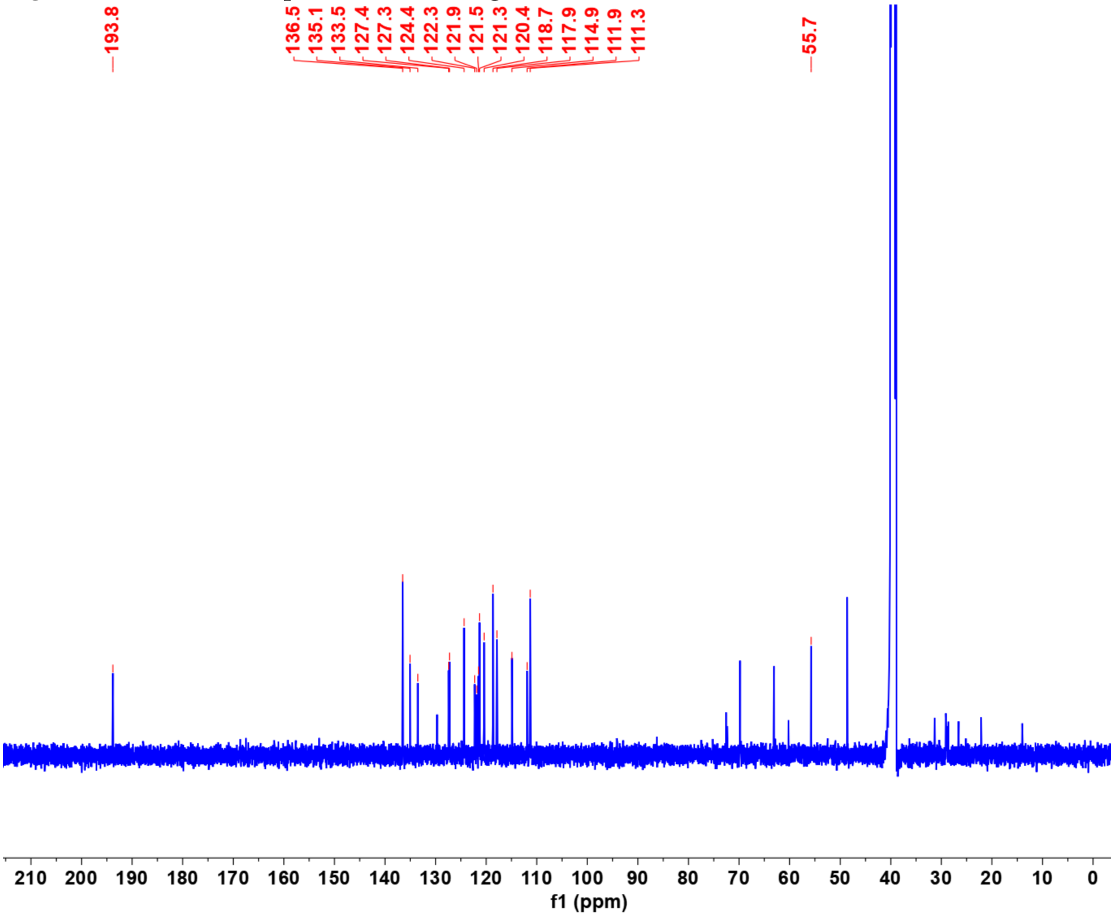

References

1. Tamura, K.; Stecher, G.; Kumar, S., MEGA11 Molecular Evolutionary Genetics Analysis Version 11. Mol. Biol. Evol. 2021, 38, (7), 3022-3027.
